# The biomechanical properties of an epithelial tissue determine the location of its vasculature

**DOI:** 10.1038/ncomms13560

**Published:** 2016-12-20

**Authors:** Martin Kragl, Rajib Schubert, Haiko Karsjens, Silke Otter, Barbara Bartosinska, Kay Jeruschke, Jürgen Weiss, Chunguang Chen, David Alsteens, Oliver Kuss, Stephan Speier, Daniel Eberhard, Daniel J. Müller, Eckhard Lammert

**Affiliations:** 1 Institute of Metabolic Physiology, Department of Biology, Heinrich Heine University, D-40225 Düsseldorf, Germany; 2 German Center for Diabetes Research (DZD e.V.), D-85764 München-Neuherberg, Germany; 3 Institute for Beta Cell Biology, German Diabetes Center, Leibniz Center for Diabetes Research at Heinrich Heine University, D-40225 Düsseldorf, Germany; 4 Eidgenössische Technische Hochschule Zürich, Department of Biosystems Science and Engineering, CH-4058 Basel, Switzerland; 5 Institute for Clinical Biochemistry and Pathobiochemistry, German Diabetes Center, Leibniz Center for Diabetes Research at Heinrich Heine University, D-40225 Düsseldorf, Germany; 6 Paul Langerhans Institute Dresden (PLID) of Helmholtz Center Munich at the University Clinic Carl Gustav Carus of Technische Universität Dresden, Helmholtz Zentrum München, D-85764 Neuherberg, Germany; 7 DFG-Center for Regenerative Therapies Dresden (CRTD), Faculty of Medicine, Technische Universität Dresden, D-01307 Dresden, Germany; 8 Institute for Biometrics and Epidemiology, German Diabetes Center, Leibniz Center for Diabetes Research at Heinrich Heine University, D-40225 Düsseldorf, Germany

## Abstract

An important question is how growing tissues establish a blood vessel network. Here we study vascular network formation in pancreatic islets, endocrine tissues derived from pancreatic epithelium. We find that depletion of integrin-linked kinase (ILK) in the pancreatic epithelial cells of mice results in glucose intolerance due to a loss of the intra-islet vasculature. In turn, blood vessels accumulate at the islet periphery. Neither alterations in endothelial cell proliferation, apoptosis, morphology, *Vegfa* expression and VEGF-A secretion nor ‘empty sleeves' of vascular basement membrane are found. Instead, biophysical experiments reveal that the biomechanical properties of pancreatic islet cells, such as their actomyosin-mediated cortex tension and adhesive forces to endothelial cells, are significantly changed. These results suggest that a sorting event is driving the segregation of endothelial and epithelial cells and indicate that the epithelial biomechanical properties determine whether the blood vasculature invades or envelops a growing epithelial tissue.

## Abstract (web summary)

Vasculature is denser in soft than in stiff tissues. Kragl *et al*. suggest a mechanistic link between biomechanical tissue properties and vascularization by showing that integrin-linked kinase reduces the contractile forces of the cell cortex in endocrine pancreatic cells, facilitating their adhesion to blood vessels and enabling pancreatic islet vascularization.

## Graphical abstract

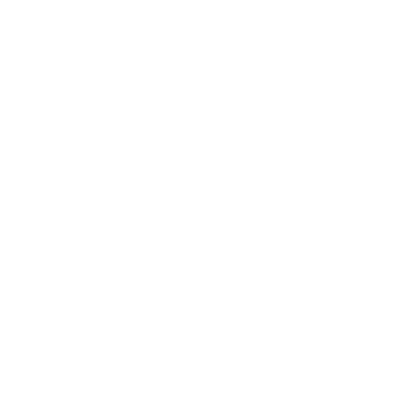

Formation and maintenance of a blood vessel network has a key role both during development and disease^1^,^2^. In pancreatic islets, a dense network of blood capillaries contributes to glucose homeostasis by transporting blood glucose to and insulin (the key blood glucose-lowering hormone) from pancreatic beta cells (the major endocrine cell type in pancreatic islets)^3^. The beta cells interact with blood vessels via the vascular basement membrane that surrounds the islet capillaries^4^. Several *in vitro* studies on rodent and human islets, pancreatic beta cells, and pancreatic epithelium provided evidence that their integrins bind to basement membranes and endothelial cell-derived factors to facilitate beta cell differentiation, proliferation, and function^5^,^6^,^7^. Some, but not all, *in vivo* studies also support a role of integrins in beta cell proliferation and function^8^,^9^,^10^,^11^. Notably, integrin-linked kinase (ILK) binds to the cytoplasmic tails of integrins expressed in pancreatic islets^12^.

Here we investigated the role of ILK in islet endocrine cells *in vitro* and *in vivo* and found that knockdown of *Ilk* in mouse insulinoma cells and deletion of *Ilk* in the pancreatic epithelium of mice reduce the adhesion strength of the endocrine cells to a vascular endothelial cell line, while at the same time increase cortex tension of the endocrine cells. The latter findings help to explain why deletion of *Ilk* in pancreatic epithelium leads to a loss of the intra-islet vasculature and excessive accumulation of the vasculature at the islet periphery. Notably, the sum of intra- and peri-islet vascular endothelial cells was unchanged, no ‘empty sleeves' of vascular basement membrane were observed during the onset of this vascular phenotype, and endothelial cell proliferation, apoptosis and morphology as well as secretion of vascular endothelial growth factor-A (VEGF-A) were not altered. The data suggest a mechanical sorting event, rather than a chemotactic one in response to angiogenic growth factors, driving the segregation of vascular endothelial cells and *Ilk*-deficient endocrine cells during pancreatic islet growth.

## Results

### Requirement of *Ilk* for normal insulin secretion *in vivo*

**Figure 1:**
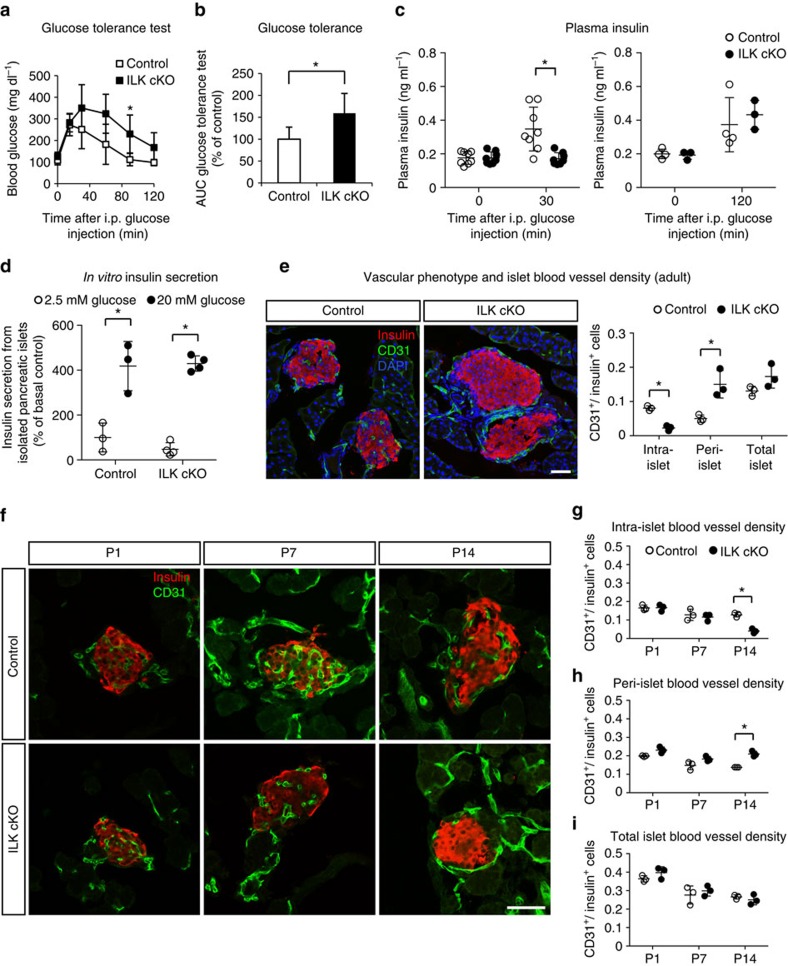
ILK in pancreatic islets is required for a normal localization of their vasculature. (**a**) Blood glucose concentrations during a glucose tolerance test in male ILK cKO mice (filled squares) and heterozygous littermate controls (unfilled squares) at the age of 7 weeks. Glucose (1 g kg^−1^ body weight) was injected i.p. at 0 min. *N*=7–8 mice per experimental group. (**b**) Area under curve of data shown in **a**. (**c**) Plasma insulin concentrations in male ILK cKO and control mice (10-weeks-old). Glucose (1 g kg^−1^ body weight) was injected i.p. at 0 min. *N*=8 mice per experimental group for 0 and 30 min (left panel), and *N*=3–4 mice per group for 0 and 120 min (right panel). (**d**) Insulin secretion from isolated pancreatic islets of ILK cKO and control mice at 2.5 mM (white dots) or 20 mM (black dots) glucose. Secreted insulin is normalized to total insulin levels. *N*=3–4 islet batches each. (**e**) Laser scanning microscopy (LSM) images of immunofluorescence staining for insulin (a marker for pancreatic beta cells) and CD31 (a marker for blood vessels) of pancreatic sections from 12-weeks-old control and ILK cKO mice. Quantification of intra-islet, peri-islet and total islet blood vessel densities in ILK cKO and control mice. *N*=3 animals per experimental group, 8–10 islets were analysed per animal. (**f**) LSM images of immunofluorescence staining for insulin and CD31 in pancreatic sections from 1 day (P1), 7 days (P7) and 14 days (P14) old control and ILK cKO mice. (**g**–**i**) Quantification of (**g**) the intra-islet vascular density, (**h**) the peri-islet vascular density and (**i**) the total islet vascular density, each relative to the insulin^+^ pancreatic beta cell number. *N*=3 animals per experimental group with 28–39 (P1), 53–114 (P7) and 47–77 (P14) islets analysed per animal. Data are shown as mean values±s.d., *P<0.05 in an unpaired, two-sided Student's *t*-test with (**a**) Holm-Bonferroni correction. Scale bars, 50 μm (**e**,**f**).

First, we specifically deleted *Ilk* in the pancreatic epithelium by generating pancreas-duodenum homeobox 1 *(Pdx1)-Cre; Ilk*^*loxP/loxP*^ mice (referred to as ILK cKO hereafter) (Supplementary Fig. 1). In adult mice, a strong reduction of *Ilk* messenger RNA (mRNA) and protein expression was observed in ILK cKO islets compared with those of heterozygous control islets (Supplementary Fig. 1a,b). The ILK cKO mice appeared to be normal in their fasting blood glucose concentrations, but exhibited a reduced glucose tolerance when challenged in an intraperitoneal glucose tolerance test (Fig. 1a,b). Moreover, after an intraperitoneal glucose injection, plasma insulin concentrations failed to rise in ILK cKO mice at 30 min post injection, but did rise after 120 min, indicating a delayed insulin secretion from pancreatic islets (Fig. 1c). In contrast, insulin tolerance remained normal (Supplementary Fig. 1c,d).

Next, we measured insulin secretion from isolated control and ILK cKO islets under low (2.5 mM) and high (20 mM) glucose concentrations *in vitro*. To our surprise, and in contrast to the *in vivo* situation, virtually no reduction of glucose-stimulated insulin secretion from *Ilk*-deficient islets was observed *in vitro* when compared with control islets (Fig. 1d). Further, ILK cKO islets had a normal insulin content and also responded to an increase in glucose concentration from 5 to 10 mM with enhanced insulin release (Supplementary Fig. 1e,f).

### Mislocalized pancreatic islet vasculature in ILK cKO mice

To explain the discrepancy between the *in vitro* and *in vivo* situation, we next investigated whether the *in vivo* failure of ILK cKO mice to rapidly increase plasma insulin concentrations upon glucose injection might have been caused more by a failure of the ILK cKO islets to deliver insulin into the blood stream than a failure of these islets to secrete insulin. The islet capillary network enables glucose tolerance by distributing insulin from islets to peripheral tissues^13^,^14^,^15^; thus, we analysed islet vascular networks in the pancreas from ILK cKO and heterozygous control mice (Fig. 1e). Notably, immunohistochemical staining of pancreatic sections from adult mice for the endothelial cell marker CD31 revealed a striking defect in vascular organization of ILK cKO islets (Fig. 1e), implicating a possible reason for the failure of ILK cKO mice to control blood glucose concentrations (Fig. 1a,b).

An ∼80% reduction of intra-islet capillaries was observed in ILK cKO islets (Fig. 1e), while no signs of increased hypoxia were noted (Supplementary Fig. 2). In contrast, significantly more blood vessels were found at the islet periphery (Fig. 1e). Notably, the total number of islet endothelial cells, or the sum of intra- and peri-islet endothelial cells, did not significantly differ between control and ILK cKO islets (Fig. 1e; Supplementary Movies 1 and 2). Moreover, a similar vascular phenotype was observed when the *Ilk* gene was deleted using a previously published beta cell-specific *Ins1(Cre)* knock-in mouse^16^ (Supplementary Fig. 3). Further, after transplantation of ILK cKO and control islets into the anterior eye chamber of mice, a dense intra-islet vascular network was found in control islets 28 days post transplantation, whereas the intra-islet blood vessel density was reduced in ILK cKO islets (Supplementary Fig. 4a). To further describe the glucose intolerance and delayed insulin release observed in ILK cKO mice, we investigated whether the islet capillaries were perfused. After injection of a fluorescein isothiocyanate (FITC)-labelled tomato lectin into the tail vein of mice, all analysed islet vessels were coated with the fluorescent lectin (Supplementary Fig. 4b), indicating that the blood vessels in ILK cKO islets were perfused despite their altered localization.

### Postnatal onset of the vascular phenotype in ILK cKO mice

To gain more insight into how ILK in islet endocrine cells affects the intra-islet vasculature, we analysed the age at which changes in the islet vasculature were first detected (Fig. 1f). We noted that the mRNA for ILK was reduced in islets 7 days after birth, but more strongly reduced 14 days after birth (Supplementary Fig. 1g). Thus, we quantified islet vascular endothelial cells of newborn mice at 1, 7 and 14 days after birth (P1, P7 and P14, respectively). Islets in P1 and P7 ILK cKO mice appeared to be normal in their blood capillary distribution (Fig. 1f–i). In contrast, P14 mice displayed an islet vascular phenotype similar to the one observed in adult ILK cKO mice with depleted intra-islet, elevated peri-islet and unchanged total islet endothelial cell counts (Fig. 1f–i), showing that the vascular phenotype develops between P7 and P14.

### Normal VEGF-A secretion from ILK cKO islets

**Figure 2:**
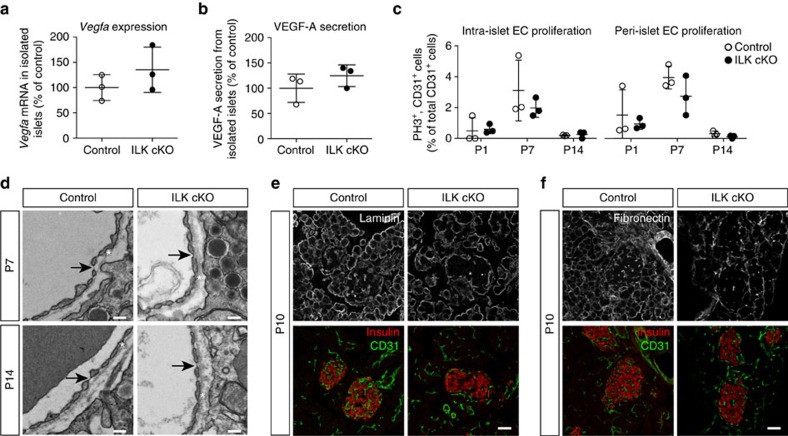
ILK in islets is required for neither endothelial cell proliferation nor survival. (**a**) Quantification of relative *Vegfa* mRNA levels in control versus ILK cKO islets. *N*=3 animals per experimental group. (**b**) Quantification of VEGF-A protein secreted from control versus ILK cKO islets. *N*=3 islet batches for each experimental condition. (**c**) Quantification of proliferating intra-islet vascular endothelial cells (left) and peri-islet endothelial cells (right). CD31^+^, phospho-histone 3 (PH3)^+^ cells are shown as percentages of the total number of intra-islet and peri-islet CD31^+^ cells, respectively, in islets of pancreatic sections from mice at the age of 1, 7 and 14 days. *N*=3 animals per experimental group. A total number of 167–337 (P1), 314–688 (P7) and 113–659 (P14) CD31^+^ intra-islet endothelial cells and a total number of 159–371 (P1), 193–607 (P7) and 441–628 (P14) CD31^+^ peri-islet endothelial cells per animal were analysed. (**d**) Transmission electron microscopy images of capillaries in a control and an ILK cKO islet. Arrows point to the fenestrations in the blood vessel wall, which are typical of islet capillaries and require VEGF-A signalling, and asterisks indicate the location of basement membrane surrounding the islet capillary. (**e**,**f**) LSM images of immunofluorescence staining for laminin or fibronectin (white), insulin (red) and CD31 (green) in pancreatic sections from 10-day-old control and ILK cKO mice. Data are shown as mean values±s.d., *P<0.05 in an unpaired, two-sided Student's *t*-test. Scale bars, 200 nm (**d**); scale bars, 50 μm (**e**,**f**).

Next, we investigated the mechanism by which ILK in endocrine, non-endothelial pancreatic cells affected islet vascularization. ILK has been shown to influence gene transcription, as well as cell proliferation, adhesion and motility, depending on the cell and tissue type in which it is expressed^12^. Thus, a number of possible scenarios could explain the novel vascular phenotype observed in the pancreatic islets upon *Ilk* deletion. As formation and maintenance of the islet vasculature strictly depends on VEGF-A^13^,^15^,^17^,^18^, we wondered whether ILK promotes islet vascularization by maintaining VEGF-A expression. Interestingly, prior studies using a few murine models of cancer demonstrated that inhibition of ILK decreased hypoxia-induced *Vegfa* gene transcription^19^,^20^. We, therefore, quantified *Vegfa* mRNA levels in control and ILK cKO islets (Fig. 2a). However, we did not observe lower expression of *Vegfa* that could explain the reduced blood vessel density in islets upon *Ilk* deletion (Fig. 2a). Likewise, we did not detect any reduction in VEGF-A secretion from isolated ILK cKO islets (Fig. 2b). Consistent with these results, ILK cKO islets did not resemble islets lacking VEGF-A, which are generally depleted of both intra-islet and peri-islet blood vessels^13^,^15^,^17^,^18^. Further, immunohistochemical staining of pancreases in the adult and at P10, the time at which the vascular phenotype starts to develop, revealed no obvious changes in the localization of VEGF-A within the islets (Supplementary Fig. 5). The data, therefore, suggest that in pancreatic islets ILK does not support vascular density by increasing expression, changing location or secretion of VEGF-A.

Apart from VEGF-A, many other pro- and anti-angiogenic growth factors are known, making it possible that ILK depletion affects another factor. For example, some, but not all studies reported that angiopoietins have a role in islet vascularization^21^,^22^,^23^. We thus quantified the mRNA levels of Angiopoietin-1 and Angiopoietin-2, but did not detect any difference between control and ILK cKO islets either (Supplementary Fig. 6a,b). Similarly, the expression of the anti-angiogenic isoforms of *Vegfa* were not different (Supplementary Fig. 6c)^24^. Since most pro- and anti-angiogenic factors affect endothelial cell survival, proliferation and morphology^1^,^2^,^13^,^15^, we next analysed these parameters in control versus ILK cKO islets. Vascular endothelial cell proliferation in ILK cKO islets was found to be only slightly and non-significantly reduced, and this tendency was observed for both intra- and peri-islet endothelial cells (Fig. 2c), thus being unable to explain the inverse change of endothelial cell number in islet core and periphery. Moreover, apoptotic endothelial cells were not detected in the islets of ILK cKO mice, neither at P7 nor at P10 (Supplementary Table 1), indicating that increased endothelial cell death is not responsible for the lack of intra-islet capillaries. Further, the ultrastructure of intra-islet capillaries, including the presence of fenestrations and vascular basement membrane, was investigated at P7 and P14 and found to be largely unchanged (Fig. 2d). In P10 and adult ILK cKO islets, blood vessels stained positive for the vascular basement membrane proteins laminin and fibronectin (Fig. 2e,f; Supplementary Fig. 7), and were covered with NG2-positive pericytes (Supplementary Fig. 8). In addition, the staining of these basement membrane proteins and NG2 overlapped with the CD31 staining of endothelial cells (Fig. 2e,f; Supplementary Figs 7 and 8). Based on these results, it seemed unlikely that mainly alterations in pro- or anti-angiogenic growth factor expression and secretion accounted for the striking vascular phenotype observed.

### ILK cKO mice show no change in beta cell proliferation

Another possible mechanism to explain the phenotype could reside in increased endocrine cell proliferation upon *Ilk* deletion, as the deletion of *Ilk* was recently reported to increase hepatocyte proliferation^25^. We thus asked whether a ‘dilution' of vascular endothelial cells, caused by an increased endocrine pancreatic cell proliferation, could account for the reduced intra-islet vascular density observed upon *Ilk* deletion. However, analysis of the average number of beta cells per islet did not reveal any difference between islets from ILK cKO animals versus control littermates (Supplementary Fig. 9a). Similarly, neither the adult beta cell mass nor the proliferation rate of beta cells in the newborn pancreas changed upon *Ilk* deletion (Supplementary Fig. 9b,c). Therefore, the vascular phenotype cannot be explained with an increased endocrine to endothelial cell number within the pancreatic islets.

### Failure of ILK cKO islets to adhere to basement membrane

Searching for a reason behind the observed vascular phenotype, we considered its postnatal manifestation. During mammalian embryonic development, endocrine cells derive from pancreatic epithelium and vascular endothelial cells aggregate to form highly vascularized islet cell clusters^3^. After birth, these islet cell clusters expand along with their vasculature, undergo morphogenetic changes, and mature into glucose-responsive, vascularized pancreatic islets^26^. While islet vascularization appeared to be normal in the islets of newborn mice, the observed vascular phenotype is established during postnatal islet growth. The separation of endocrine pancreatic cells and vascular endothelial cells during that time reminded us of cell sorting events, such as the segregation of cell lineages during zebrafish gastrulation or pattern formation during embryonic development^27^,^28^,^29^. A prominent explanation of these cell sorting events is the modified Steinberg's differential adhesion hypothesis, proposing that different tissues act like liquids whose different degrees of surface adhesion cause them to either adhere or separate from each other^30^,^31^. Since several studies showed that ILK promotes cell–cell and cell–matrix adhesion during developmental processes and pathogenesis^32^,^33^,^34^,^35^,^36^,^37^, we hypothesized that during postnatal growth, ILK in endocrine pancreatic cells facilitates their adhesion to blood vessels to prevent the vascular endothelium from segregating from the pancreatic islet.

**Figure 3:**
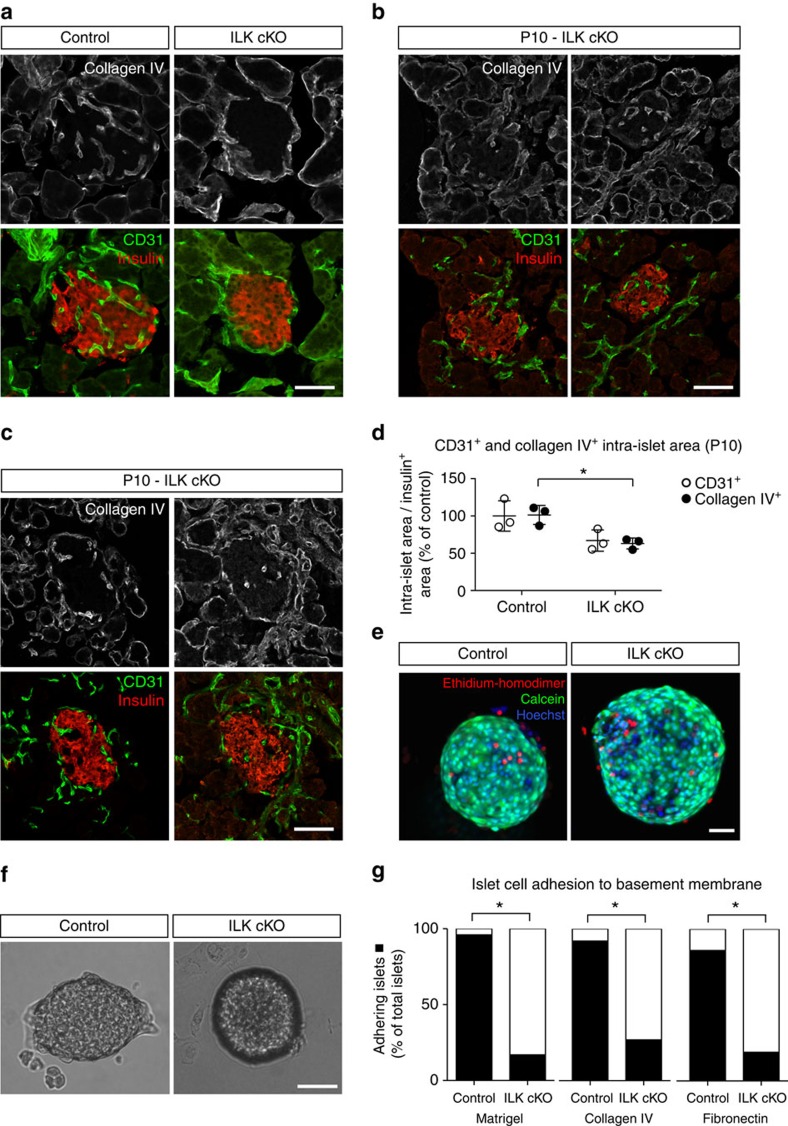
Reduced endocrine pancreatic cell adhesion to basement membrane components. (**a**–**c**) LSM images of immunofluorescence staining for CD31 (green), insulin (red) and collagen IV (a basement membrane protein shown in white) of pancreatic sections from 3-weeks control and ILK cKO mice (**a**) and 10-days-old ILK cKO mice (P10) (**b**,**c**). (**d**) Quantification of the intra-islet CD31 and collagen IV positive areas within control and ILK cKO islets of 10-days-old mice. *N*=3 animals per experimental group. (**e**) Live cell imaging of a representative control and ILK cKO islet cultured for 7 days. Living cells were stained with calcein (green), dead cells with ethidium homodimer-1 (red), and all cell nuclei with Hoechst (blue). (**f**) Representative control and ILK cKO islets 7 days after plating on fibronectin. (**g**) Summary of control and ILK cKO islets adhering to matrigel, collagen IV and fibronectin. *N*=25 control and 35 ILK cKO islets for matrigel, *N*=48 control and 41 ILK cKO islets for collagen IV, and *N*=22 control and 16 ILK cKO islets for fibronectin. Data are shown as mean values±s.d., *P<0.05 in a two-way ANOVA followed by Tukey's multiple comparison test (**d**), and in a Fisher's exact test (**g**). Scale bars, 50 μm.

Since blood vessels are surrounded by a basement membrane mainly deposited by vascular endothelial cells, we first studied whether ILK depletion in endocrine pancreatic cells reduces their adhesion to the endothelial basement membrane. This hypothesis implies that blood vessels and their vascular basement membrane separate from the islet core simultaneously, leaving behind no ‘empty sleeves' characteristic of blood vessel regression usually observed upon deprivation of angiogenic growth factors^17^. To our knowledge, the latter phenotype has not yet been described during vascular regression, be it from an organ or a tumour^17^,^38^. To study this scenario, pancreatic sections were stained for CD31 and collagen IV (a component of the vascular basement membrane). As with vascular endothelial cells, there was a significant reduction of collagen IV in ILK cKO islets versus control islets (Fig. 3a). Notably, no ‘empty sleeves' of vascular basement membrane were observed^17^,^38^; that is, no basement membrane was found in the islet that did not co-localize with an endothelial cell (Fig. 3a). The latter observation also holds true for pancreatic islets at P10 where the vascular phenotype just starts to establish, as seen in four images taken from pancreatic islets at P10 and stained for CD31, collagen IV and insulin (Fig. 3b–d). Compared with control islets, ILK cKO islets presented an ∼40% reduction of the blood vessel and collagen IV area at P10 with no difference observed between the CD31 and collagen IV areas in ILK cKO islets (Fig. 3d). Therefore, ILK might be required to provide endocrine pancreatic cells with sufficient adhesive strength to prevent the vasculature with its basement membrane from separating out of the islet.

To directly test whether ILK-depleted islets differed from control islets in their adhesion strength, islets were plated on a reconstituted basement membrane (that is, growth factor-deprived matrigel) as well as two basement membrane components (that is, collagen IV and fibronectin). After showing that ILK-depleted islets did not significantly differ from control islets in their cell survival upon cultivation (Fig. 3e), islets were plated and incubated for 7 days (Fig. 3f). Whereas the majority of control islets adhered to the basement membrane components, most ILK cKO islets failed to do so (Fig. 3f,g; Supplementary Movies 3 and 4), supporting the notion that pancreatic islet cells fail to adhere to the basement membrane of the blood vasculature as a possible reason for the segregation of islets and blood vessels.

### ILK cKO cells poorly adhere to endothelial cells

**Figure 4:**
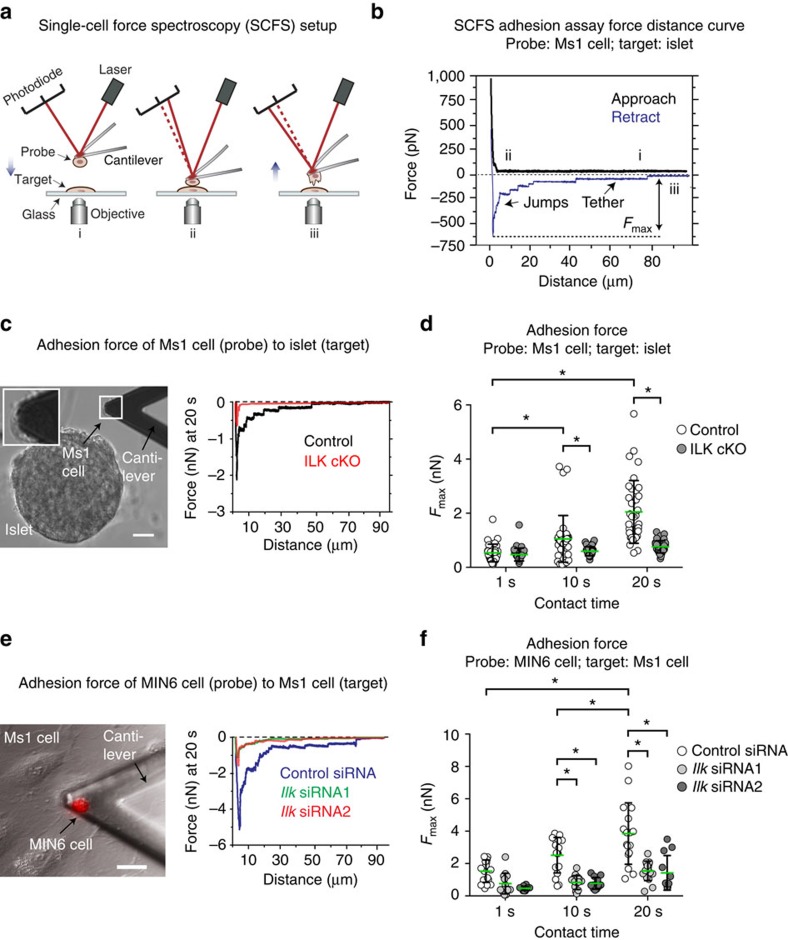
Endocrine pancreatic cells require ILK for adhering to vascular endothelial cells. (**a**) Outline of a single-cell force spectroscopy (SCFS) experiment. A single cell or islet ‘probe' immobilized on an atomic force microscopy (AFM) cantilever is approached into contact with a ‘target' cell or islet (i). After a predefined contact time (ii), the probe was retracted at 5 μm s^−1^, and adhesive forces were detected recording the cantilever deflection over the distance travelled by the cantilever (iii). (**b**) The force-distance curve records the maximum adhesion force (*F*_max_) between the probe (Ms1 endothelial cells) and target (pancreatic islets). (**c**) Representative image and adhesion force curves between Ms1 vascular endothelial cells (probe; see the inset in the image) and control (black) versus ILK cKO (red) islets. (**d**) Adhesion forces between Ms1 cells (probe) and mouse pancreatic islets (target). *N*=36 different Ms1 cells versus 36 different control pancreatic islets and 34 different Ms1 cells versus 34 different ILK cKO pancreatic islets were tested. The green lines indicate average values. (**e**) Representative image and adhesion force curves between MIN6 cells (probe; a MIN6 cell stained in red) and Ms1 microvascular endothelial cells (target). (**f**) Adhesion forces measured between MIN6 cells (probes), treated with either control or two *Ilk* siRNAs, and Ms1 endothelial cells as targets. *N*=12–17 MIN6 cells versus *N*=12-17 different Ms1 cells were tested per experimental condition (contact time and siRNA treatment). The green lines indicate average values. Data are shown as mean values±s.d., *P<0.05 in a two-way ANOVA followed by Tukey's multiple comparison test. Scale bars, 15 μm (**c**,**e**).

Next, we used an atomic force microscope (AFM) to perform single-cell force spectroscopy (SCFS) (Fig. 4). This technique allows the measurement of adhesive forces between two single cells or tissues, as well as a single cell or tissue and a substrate (Fig. 4)^39^. Briefly, SCFS uses a cantilever on which the cell or tissue probe is immobilized and then brought into contact with an adherent cell or a substrate. Adhesive forces are measured by the retraction of the cantilever after a pre-defined contact time in a range of several seconds or minutes. The adhesion strength is measured by the retraction-induced deflection of the cantilever (Fig. 4a,b)^39^,^40^,^41^.

First, we quantified the adhesive forces between vascular endothelial cells and whole pancreatic islets (Fig. 4b), with either control or ILK cKO endocrine pancreatic cells (Fig. 4c). For this experiment, we used Ms1 cells, which are immortalized pancreatic islet microvascular endothelial cells that produce vascular basement membrane components, including collagen IV (ref. 42). Notably, a lower adhesion strength was observed between ILK cKO pancreatic islets and Ms1 endothelial cells compared with control islets and Ms1 cells (Fig. 4c,d), in particular at a contact time of 20 s. The latter finding indicates a defective time-dependent strengthening of the adhesion between the pancreatic islets and vascular endothelial cells when ILK is absent from the endocrine pancreatic cells.

Second, since islets consist of different cell types besides endocrine pancreatic cells, including those in which *Ilk* was not deleted using the *Pdx1* promoter (such as fibroblasts or Schwann-like cells), we next tested whether similar or even stronger effects could be observed by replacing the islets with immortalized mouse pancreatic beta cells (that is, MIN6 cells)^43^ in which we silenced the *Ilk* transcripts using short interfering RNA (siRNA) (Fig. 4e; Supplementary Fig. 10a). Consistent with our observations in whole pancreatic islets, we observed a time-dependent difference in the adhesion strength between *Ilk*-silenced MIN6 epithelial cells and Ms1 endothelial cells (Fig. 4e,f). That is, silencing *Ilk* reduced adhesion strength between pancreatic beta cell and endothelial cell lines. Similar results were reproduced when control- and *Ilk*-silenced MIN6 cells were separated from matrigel and collagen IV (Supplementary Fig. 10b,c). As a positive control, the gene for β1 integrin (*Itgb1*) was silenced in MIN6 cells (Supplementary Fig. 11a), and this also reduced the adhesion of MIN6 cells to Ms1 endothelial cells (Supplementary Fig. 11b).

### High cortex tension in ILK cKO pancreatic islet cells

Our findings suggest that the segregation between ILK-depleted islets and their blood vasculature was due to defects in endocrine pancreatic cell adhesion to blood vessels. However, we are aware that Steinberg's hypothesis was modified soon after its release^44^, since the binding energy of adhesion molecules is not sufficient to account for cell sorting events^45^,^46^,^47^,^48^,^49^. Recent experimental and theoretical studies have supported the hypothesis that differential cell cortex tension along with differential cell adhesion drive the segregation of cell populations^28^,^50^,^51^,^52^. In different experimental set-ups, an elevated cortical actomyosin contraction has been shown to increase the cell cortex tension^53^,^54^,^55^. Since ILK has been reported to suppress actomyosin-mediated cell contractility^12^,^34^,^35^, we asked whether ILK might reduce cortex tension in pancreatic endocrine cells, thus contributing to a better adhesion of these epithelial cells to the vascular endothelial cells.

**Figure 5:**
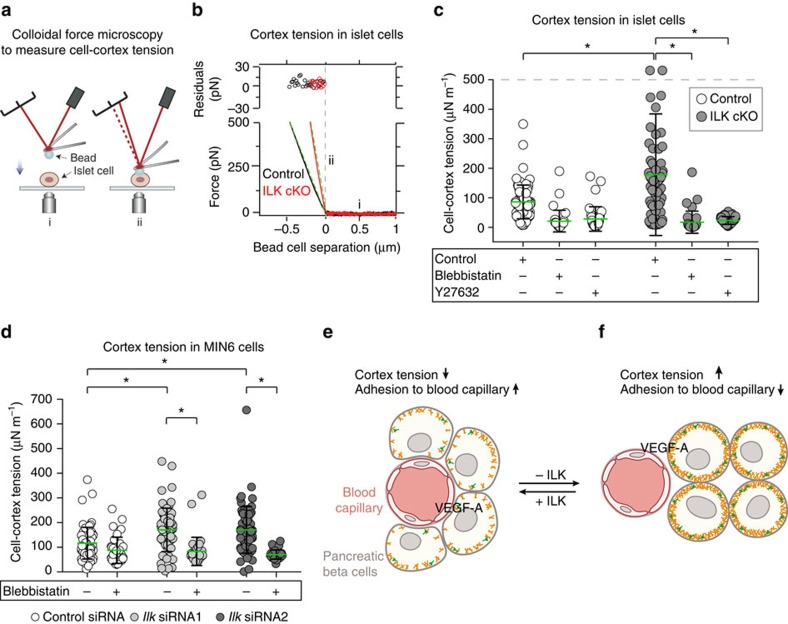
ILK regulates cortex tension via actomyosin in endocrine pancreatic cells. (**a**) Scheme to illustrate the principle of cortex tension experiment. A passivated bead attached to a tipless AFM cantilever is moved towards the endocrine pancreatic cell surface at 1 μm s^−1^ (i) and the surface is deformed by the bead (ii). (**b**) Representative force curves used for measuring cell cortex tension in control versus ILK cKO pancreatic islet cells. Cells are shown and fitted to a linear model to extract the cell cortex tension. The top panel shows the residuals of the fit. (**c**) Cortex tension in control and ILK cKO islet cells in the presence or absence of blebbistatin (a myosin II inhibitor) or Y27632 (a ROCK inhibitor). *N*=35–74 islet cells derived from *N*=5–7 islets were tested for each condition. Note that two values with 921 and 1,509 were above 500 μN m^−1^ and are depicted above the grey line. The green lines indicate average values. (**d**) Cortex tension in MIN6 cells treated with either a control or two *Ilk* siRNAs, with and without blebbistatin treatment before the measurement. *N*=35–68 cells were tested per experimental condition. (**e**,**f**) Schematic overview of pancreatic beta cells and a blood capillary in a pancreatic islet. In a control background (**e**) pancreatic beta cells surround the blood capillary. The cortex is composed of F-actin filaments (orange) and non-muscular myosin II (green). *Ilk*-deficient beta cells (**f**) display an elevated cortex tension due to an enhanced ROCK-dependent actomyosin contraction. As a result of their increased cell cortex tension and decreased cell adhesion, the endocrine cells segregate from vascular endothelial cells, and the pancreatic islet becomes avascular despite unchanged levels of VEGF-A expression and secretion. Data are shown as mean values±s.d., *P<0.05 in a two-way ANOVA followed by Tukey's multiple comparison test. Due to deviations from normality two sensitivity analyses (a quantile regression model for the median and robust regression model) were performed following the idea of Huber's M-estimation. Results were essentially unchanged in comparison to the parametric models.

Since non-muscle myosin II isoforms are expressed in endocrine pancreatic cells^56^, and since, to our knowledge, the role of ILK in cell cortex tension has not yet been studied, we measured cortical tension in control versus ILK-silenced islet cells. To this end, we deformed the surface of dissociated pancreatic endocrine cells with a colloidal force probe and recorded force-indentation curves required for deformation (Fig. 5a,b)^28^. On average, we recorded a twofold increase in cortex tension in the ILK cKO pancreatic islet cells compared with control cells (Fig. 5c). Consistent with this finding, a significantly higher cell cortex tension was also observed in ILK-depleted (but not β1 integrin-depleted) MIN6 cells versus control-treated cells (Fig. 5d; Supplementary Fig. 11c).

To test whether the elevated cortex tension observed upon ILK depletion required actomyosin contraction, we treated control- and ILK-depleted pancreatic islet and MIN6 cells with blebbistatin, a widely used inhibitor of myosin II^28^,^57^. We also applied Y27632, a frequently applied inhibitor of Rho-associated protein kinase (ROCK)^58^, which was previously shown to be downstream of ILK^34^,^35^. The levels of cortex tension were reduced by both treatments, and no longer significantly differed between control and ILK-depleted islet cells (Fig. 5c,d), suggesting that actomyosin contraction significantly contributed to the increased cell surface tension observed in ILK-depleted endocrine pancreatic cells. Furthermore, phalloidin staining of control pancreatic islets cultured on fibronectin revealed a reorganization of their cortical actin cytoskeleton towards the basement membrane (Supplementary Fig. 12a). In stark contrast, this kind of reorganization was not observed in ILK cKO pancreatic islets (Supplementary Fig. 12a; Supplementary Movies 3 and 4), consistent with the observed increase in actomyosin-mediated cortex tension, which has been reported to make cells more rigid and less compliant^59^. To directly test whether the increased actomyosin contraction in ILK-depleted endocrine pancreatic cells was required to reduce their adhesion to vascular endothelial cells, we dissociated control and ILK cKO pancreatic islets, treated them with blebbistatin or Y27632 and performed SCFS (Supplementary Fig. 12b). Notably, upon blebbistatin or Y27632 treatment, similar forces were needed to separate ILK cKO pancreatic islet cells and control islet cells from Ms1 cells, showing that lowering the actomyosin contraction rescued the adhesion of ILK-depleted endocrine pancreatic cells to vascular endothelial cells (Supplementary Fig. 12b). Similar results were obtained when MIN6 cells were treated with two different ROCK inhibitors (Supplementary Fig. 12c).

## Discussion

Our results support the hypothesis that limiting the contractile forces of the cell cortex is an integral part of epithelial cell adhesion to blood capillaries. Mechanistically, we propose that ILK reduces these forces in endocrine pancreatic cells by inhibiting non-muscle myosin II, via inhibition of ROCK, to facilitate their adhesion to blood vessels and thus enable pancreatic islet vascularization (Fig. 5e). Notably, previous studies have shown that ILK regulates myosin II via various molecular mechanisms, including RhoA/ROCK and calcium regulatory protein sarcoplasmic/endoplasmic reticulum Ca^2+^ ATPase isoform-2a (SERCA-2a) and phospholamban^34^,^60^. While our study does not exclude a role for soluble angiogenic factors in ILK cKO islets (Fig. 5e), our observations strongly favour the interpretation that the altered biomechanical properties of the endocrine tissue prompted the observed vascular phenotype (Fig. 5f). As shown, vascular endothelial cell number, proliferation and apoptosis are unchanged in ILK cKO pancreatic islets, contrary to what would be predicted during either upregulation or downregulation of growth factor receptor signalling; likewise, the unchanged expression and secretion of VEGF-A, the presence of endothelial fenestration and vascular basement membrane, and absence of ‘empty sleeves' of vascular basement membrane oppose the established phenotypic response to changes in angiogenic factors^17^,^61^,^62^ (Fig. 5e,f).

To date, from embryonic development to tumorigenesis, studies on tissue neovascularization have largely focused on the cellular and molecular components of the vasculature and its growth factors. However, our results motivate future pro- and anti-angiogenic studies to therapeutically target the epithelial cell mechanics of a tissue. For further consideration, after development, the biomechanical stiffness of several tissues inversely correlates with their microvascular densities (for example, compare brain and skeletal muscle)^63^; similarly, the metastatic potential of certain human cancer cells inversely correlates with both their mechanical stiffness and actomyosin contraction^59^,^64^. By stepping away from a pure endothelial-centric perspective, future studies might leverage the biomechanical properties of growing or hypertrophic tissues to inhibit or promote vascularization.

## Methods

### Mouse models

*Ilk*^*loxP/loxP*^, *Ins1(Cre)* knock-in, and *Pdx1-Cre* mice were previously described^16^,^36^,^65^. *Ilk*^loxP/loxP^ mice were crossed with *Pdx1-Cre* mice to obtain *Pdx1-Cre* x *Ilk*^loxP/wt^ (control) and *Pdx1-Cre* x *Ilk*^loxP/loxP^ (ILK cKO) mice on C57BL/6 background. Alternatively, they were crossed with *Ins1(Cre)* knock-in mice to obtain beta cell specific ILK cKO mice. For beta cell mass analysis *Cre*-negative x *Ilk*^loxP/loxP^ mice were used as control. All experiments were performed with either 1-day-old, 7-day-old, 14-day-old male/female mice or adult (8–20-weeks-old) male mice. All mice were taken from the local animal facility (Zentrale Einrichtung für Tierforschung und Tierschutzaufgaben, Medical Research School Düsseldorf), where they were kept in individual cages in a room controlled for temperature (22 °C), humidity (55%) and lighting (lights on from 6:00 hours to 18:00 hours.). Mice were fed with standard laboratory chow and water *ad libitum*. All animal experiments were approved by the local Animal Ethics Committee of the Landesamt für Natur, Umwelt und Verbraucherschutz Nordrhein-Westfalen (LANUV North-Rhine-Westfalia, Germany).

### Glucose tolerance test and plasma insulin concentrations

Glucose tolerance tests were performed on male mice after overnight fasting (16 h). Glucose was intraperitoneally (i.p.) injected at a concentration of 1 mg g^−1^ body weight, and blood glucose concentrations were measured before and 15, 30, 60, 90 and 120 min after glucose administration. Measurements were taken twice at each point of time using a Monometer Futura Glucometer (MedNet GmbH). Plasma insulin concentrations were measured before and 30 or 120 min after glucose injections using an ultra-sensitive rat insulin ELISA (Crystal Chem) in combination with an Infinite M200 NanoQuant reader (Tecan). No method of randomization was used. However, the animals of each group were chosen and placed in random order by the animal house staff. Investigators were not blinded.

### Insulin tolerance test

Insulin tolerance tests were performed on male mice after fasting (6 h). For testing insulin tolerance, 0.75 IU insulin (Berlinsulin H Normal, Berlin Chemie AG) was i.p. injected per kg body weight. Glucose concentrations were measured before and 15, 30, 60 and 90 min after insulin injection. No method of randomization was used. However, the animals of each group were chosen and placed in random order by the animal house staff. Investigators were not blinded.

### Isolation of mouse pancreatic islets

Isolation of islets from control and ILK cKO mice was performed according to a previously described protocol^66^. Briefly, Liberase TL Research Grade (Roche) was injected into P7, P14 or adult (8–20-week-old) mouse pancreata via the pancreatic duct. Pancreata were enzymatically digested for 17.5 min, and the reaction was stopped with DMEM (1 g l^−1^ glucose) (PAA Laboratories) supplemented with 15% FBS (Gibco, Life Technologies). After washing and filtering, the islets were separated from the exocrine tissue by gradient centrifugation and collected from the interphase between Histopaque-1077 (Sigma) and DMEM (1 g l^−1^ glucose). The adult islets were washed three times with DMEM supplemented with 15% (v/v) FBS, and cultured in CMRL medium containing 15% (v/v) FBS, 0.15% NaHCO_3_, 100 U ml^−1^ penicillin, 100 μg ml^−1^ streptomycin, 0.05 mM 2-mercaptoethanol (Gibco, Life Technologies) and 11.5 mM glucose (Sigma-Aldrich).

### Insulin secretion from mouse pancreatic islets

To determine insulin secretion, pancreatic islets were starved for 1 h in Krebs Ringer HEPES (KRH) buffer (15 mM HEPES, 5 mM KCl, 120 mM NaCl, 2 mM CaCl_2_, 2 mM MgCl_2_, 24 mM NaHCO_3_ and 1 mg ml^−1^ bovine serum albumin) supplemented with either 2 or 2.5 mM glucose. The same islets were first incubated with fresh KRH buffer supplemented with 2 or 2.5 mM glucose (low glucose) for 1 h, followed by a 1 h incubation with KRH buffer supplemented with 5, 10 or 20 mM glucose (high glucose). Subsequently, the islets were lysed in RIPA buffer (50 mM Tris-HCl (pH 7.4), 150 mM NaCl, 1 mM EDTA, 1 mM Na_3_VO_4_, 1 mM NaF, 0.25% Na-deoxycholate, 1% IGEPAL) to measure total insulin concentrations. Secreted and total insulin were measured using an ultrasensitive rat insulin ELISA (Crystal Chem) in combination with an Infinite M200 NanoQuant reader (Tecan). Secreted insulin was normalized to total insulin levels and presented as a percentage of insulin secretion at either 2.5 or 5 mM glucose.

For measurement of islet insulin content, total islet insulin content was normalized to total protein content which was measured by the bicinchoninic acid method (Thermo Scientific).

### Immunostaining and imaging

The whole pancreas was removed from mice, fixed in 4% paraformaldehyde and cryopreserved in 30% sucrose, embedded in Tissue-Tek optimum cutting temperature medium (Sakura). Twelve micrometre thick cryosections were made using a Microm HM560 Cryostat (Thermo Fisher). The following antibodies were used: polyclonal guinea pig anti-insulin 50 μg ml^−1^ (A0564, Dako), polyclonal rat anti-CD31 0.31 μg ml^−1^ (550274, BD Pharmingen), polyclonal rabbit anti-PH3 5 μg ml^−1^ (06-570, Millipore), monoclonal rabbit anti-cleaved caspase-3 1:200 (9664, Cell Signaling Technology), polyclonal rabbit anti-collagen IV 5 μg ml^−1^ (AB 756 P, Millipore), polyclonal rabbit anti-laminin 2.5 μg ml^−1^ (L9393, Sigma), polyclonal rabbit anti-fibronectin 5 μg ml^−1^ (AB2033, Millipore), polyclonal goat anti-VEGF 10 μg ml^−1^ (AF-493-SP, R&D), normal goat IgG 10 μg ml^−1^ (sc-2028, Santa Cruz), polyclonal rabbit anti-NG2 5 μg ml^−1^ (AB 5320, Millipore), donkey anti-guinea pig conjugated with Cy3 7.5 μg ml^−1^ (706-165-148, Jackson ImmunoResearch), donkey anti-rat conjugated with Alexa Fluor 488 10 μg ml^−1^ (A21208, Life Technologies), donkey anti-rat conjugated with Cy5 7.5 μg ml^−1^ (712-175-153, Jackson ImmunoResearch) and donkey anti-rabbit conjugated with Cy5 7.5 μg ml^−1^ (711-175-152, Jackson ImmunoResearch). Cell nuclei were stained using 4,6-diamidino-2-phenylindole (DAPI). Images were taken using laser scanning microscopy (LSM 710) coupled to an Axio Observer.Z1 microscope and the Zen 2010 software (Carl Zeiss MicroImaging GmbH). For blood vessel density analyses in adult mice, 8–10 islets from at least three different pancreatic sections were picked per animal. Insulin^+^ and CD31^+^ cells were counted with the help of Fiji (ImageJ) image analysis software^67^. The numbers of intra-islet and peri-islet CD31^+^ cells were normalized to the numbers of insulin^+^ cells per islet. For blood vessel densities in 1, 7 and 14 (P1, P7, P14) days old mice, pancreatic sections were taken in defined distances of 300 μm (P1, P7) or 600 μm (P14) resulting in at least four sections on one slide and representing 4% (P1, P7) or 2% (P14) of the dorsal and ventral pancreas. From these sections, the total number of islets (28–114 islets per animal, clusters of 10 or more insulin^+^ cells were considered as islets) was analysed for blood vessel densities, endothelial cell proliferation, beta cell proliferation and average beta cell numbers. Insulin^+^, CD31^+^ and PH3^+^ cells were counted with the help of Fiji image analyses software. CD31^+^ cells were normalized to insulin^+^ cells in islets (blood vessel densities), PH3^+^, CD31^+^ cells were normalized to the total numbers of intra- or peri-islet CD31^+^ cells (endothelial cell proliferation), and PH3^+^, insulin^+^ cells were normalized to the total numbers of insulin^+^ cells (beta cell proliferation). For analysing endothelial cell apoptosis, the total number of islets of approximately four sections per pancreas was analysed for cleaved caspase-3^+^, CD31^+^ cells. For intra-islet collagen IV area analyses, 8–10 islets from at least three different sections were analysed per animal. Insulin^+^ area was determined manually, CD31^+^ and collagen IV^+^ area were determined based on a threshold method using Fiji image analyses software. The CD31^+^ and collagen IV^+^ area was normalized to the insulin^+^ area.

For the analysis of beta cell mass, the insulin positive area was determined by applying Li thresholding using Fiji image analysis software. Total pancreatic area was determined manually on the basis of DAPI staining. Total beta cell mass was calculated by the multiplication of the pancreas weight with the ratio of insulin to total pancreatic area. For image analyses, investigators were blinded to treatments and genotypes.

### Islet adhesion to matrices

Fourteen millimeter microwells of glass bottom culture dishes (MatTek Corporation) were either incubated with growth factor-deprived matrigel, fibronectin or collagen IV (all BD Bioscience) at a concentration of 5 μg cm^−2^ (fibronectin and collagen IV), or at a 1:100 dilution in a total volume of 200 μl (matrigel). Glass bottom dishes were incubated for at least 1 h at 4 °C and washed several times before control and ILK cKO islets were plated in their normal culture medium. The islets were cultured for 7 days before attachment and spreading was assessed. Subsequently, islets were fixed in 4% paraformaldehyde for 1 day at 4 °C and stained for F-actin using phalloidin, fluorescently labelled with AlexaFluor 488 (Invitrogen, A12379) at a 1:50 dilution.

### Cell viability in control and ILK cKO islets

Cell viability was determined using the LIVE-DEAD Viability-Cytotoxicity Kit (Life Technologies). Control and ILK cKO islets were isolated and cultured in CMRL islet medium as described above. After 2 days of culture, whole islets were stained with 2 μM calcein, 4 μM ethidium homodimer-1 and Hoechst (1:1,000) in KRH buffer containing 2 mM glucose as described above. The islets were incubated for 30 min in the dark at 5% CO_2_ and 37 °C in a humidified chamber. Using an LSM 710 coupled to an Axio Observer.Z1 microscope (Carl Zeiss MicroImaging GmbH) equipped with a Plan-Apochromat 20 × /0.8 objective, LSM images were acquired as maximum intensity projections.

### Cell culture

The immortalized mouse cell line MIN6 shows many characteristics of differentiated beta cells, like insulin production and insulin secretion upon glucose stimulation^43^. MIN6 cells were cultured at 5% CO_2_ and 37 °C in a humidified chamber in DMEM supplemented with sodium pyruvate, glutamine and 4.5 g l^−1^ glucose (Gibco, Life Technologies), containing 15% heat-inactivated FBS, 0.34% sodium bicarbonate, 100 U ml^−1^ penicillin, 100 μg ml^−1^ streptomycin and 50 μM 2-mercaptoethanol (Gibco, Life Technologies). They were split when a confluency of 80–90% was reached and used for experiments at passages 20–45. Ms1 cells were cultured at 5% CO_2_ and 37 °C in a humidified chamber in DMEM supplemented with 1 g l^−1^ glucose (American Type Culture Collection), 5% FBS, 100 U ml^−1^ penicillin and 100 μg ml^−1^ streptomycin. They were split at 80–90% confluency and used for experiments at passages 4–48. All cell lines were regularly tested for mycoplasma infection using Mycoplasmacheck (GATC Biotech AG).

### siRNA transfection

For each siRNA transfection, a confluent T75 flask (Sarstedt) of MIN6 cells was electroporated using an Amaxa biosystems nucleofector II and an Amaxa cell line nucleofection kit (Lonza, VCA-1003). For *Ilk* knockdown, two different stealth siRNAs (ILKMSS205468, ILKRSS301330, Invitrogen) directed against two different regions of the *Ilk* mRNA were used at a final concentration of 800 nM for knockdown experiments. A stealth siRNA negative control medium-GC was used as negative control. For *Itgb1* knockdown, two different siRNAs (5′-CCACAGAAGUUUACAUUAA-3′ sense, 5′-UUAAUGUAAACUUCUGUGG-3 antisense; 5′-CGGAUUUGAUGAAUGAAAU-3′ sense, 5′-AUUUCAUUCAUCAAAUCCG-3′ antisense, Eurogentec) directed against two different regions of the *Itgb1* mRNA were used at a final concentration of 800 nM for knockdown experiments. An siRNA with equivalent GC content was used as control. The knockdown efficiency was confirmed 72 h post transfection using real-time reverse transcription (RT)–PCR, and experiments were performed at that time point.

### Adhesion measurements

Cell adhesion measurements were conducted with an AFM (CellHesion II, JPK Instruments) mounted on an inverted fluorescence microscope (Zeiss Axiovert 200, equipped with 20 × objective) used in closed height feedback mode^39^. Differential interference contrast imaging was used to monitor cellular morphology, and fluorescence imaging used to select cells transfected with siRNA constructs during adhesion measurements. Each tipless AFM cantilever (NPO-010, Bruker) was calibrated three times using the thermal noise to eliminate errors. Spring constants were within 10% of the nominal value (∼60 mN m^−1^). Plasma-activated cantilevers were incubated with 2.5 mg ml^−1^ Concanavalin A (ConA, Sigma) overnight at 4 °C and carefully rinsed in phosphate buffered saline (PBS) before use. All ‘probe' cells were washed with PBS, detached using 1% (vol) ethylenediaminetetraacetic acid (EDTA, Sigma), and placed into glass bottomed 35 mm diameter petri dishes (WPI Inc.) in the appropriate medium to be picked for SCFS experiments. All SCFS measurements were carried out at 37 °C using the JPK petri dish heater (JPK, Germany) and in 5% CO_2_ in air. The gas was first humidified to at least 95% of humidity using a sense silicone membrane (Permselect, Michigan, USA). The humidity level was monitored using a sensor (sensirion, Switzerland). The humidified gas was perfused on the top of the petri dish using the JPK perfusion system (JPK, Germany). The ConA-coated cantilever was gently pressed onto a cell applying a force of ∼2 nN for ∼3 s. After this, the cantilever was lifted for 2−10 min to allow the cell to attach firmly to the cantilever. This ‘probe-cell' was then moved above a ‘target-cell' or an extracellular matrix (ECM) protein covalently attached to the substrate. Cell adhesion experiments between probe-cell and functionalized surface or target-cell were performed applying a contact force of ∼1 nN, contact times ranging from 1−20 s, and ∼5 μm s^−1^ approach and retract velocities. The contact time was varied randomly for a given cell−substrate or cell–cell couple to prevent systematic bias or history effects. Each force−distance (F−D) curve characterizing the adhesion between probe and target was repeated depending on the contact time: 1 s contact time, 5 repetitions; 10 s contact time, 3 repetitions, 20 s being measured twice and 5 min being measured once. A resting time of 30 s was given between recording each force-distance curve. Each probe-cell was used to test several target-cells and different regions within functionalized surfaces with ECM proteins. One force-distance curve was taken with any given probe-cell. Cells were observed continuously during the SCFS experiment to judge whether they were intact and stably associated with the cantilever/substrate. force-distance curves were analysed using JPK analysis software to extract maximum adhesion force and cell deformation during cell−cell and cell–substrate contact. force-distance curves were pooled and statistically processed as described (see Statistical Analysis). To alter cell adhesion, cells were treated with 50 μM blebbistatin (Merck Millipore, 203389), 50 μM Y27632 (Merck Millipore, 688001) or 10 μM H1152 (Merck Millipore, 555552) with the exception of islet cells, which were treated with 55 μM blebbistatin or 55 μM Y27632, respectively.

### Functionalized surfaces

Round glass coverslip of a diameter of 4 mm were coated by electron beam thermal evaporation with a 5 nm thick aluminum layer followed by a 10 nm thick gold layer. Gold coated surface were cleaned with ethanol, dried using a gentle flow of N_2_ and cleaned by ultraviolet-ozone for 20 min. Surfaces were then immersed for 14 h in 1 mM of 16-mercaptohexadecanoic acid (5%) and 11-mercapto-1-undecanol (95%) and rinsed in ethanol. The self-assembled monolayers were immersed for 30 min into a 170 mM N-hydroxysuccinimide (NHS, Sigma) and 260 mM 1-ethyl-3-(3-dimethylaminopropyl)-carbodiimide (EDC, Sigma) and rinsed with water. The activated surfaces were then incubated with 200 μg ml^−1^ of ECM (for example, Fibronectin) in PBS for 1 h at 37 °C and rinsed with PBS. Glass coated substrates were then transferred to a petri dish and fixed using a small piece of adhesive tape.

### Cell-cortex tension measurements

Cortex tension measurements with colloidal force microscopy were carried out as described previously^28^. Briefly, an AFM cantilever was modified with a 5 μm diameter glass bead (Kisker) and coated with heated-inactivated FCS (Invitrogen) to prevent unspecific binding to the target-cell during contact measurement. The colloidal force probe was brought into contact with the cell with a 500 pN contact force at 1 μm s^−1^. A fit to the cortical shell liquid core model between 125 and 250 pN yielded cortex tension. To alter cortex tension, cells were treated with 50 μM blebbistatin (Merck Millipore, 203389) or 50 μM Y27632 (Merck Millipore, 688001) with the exception of islet cells, which were treated with 55 μM blebbistatin.

### RNA isolation and real-time RT–PCR

Total RNA was extracted from MIN6 cells and isolated mouse pancreatic islets using peqGold Trifast (Peqlab). In all, 0.2 μg (islets) or 0.4 μg (MIN6 cells) of purified RNA was reverse transcribed into complementary DNA using Superscript II reverse transcriptase (Invitrogen) with random primers according to the protocol provided by the manufacturer. Real-time RT–PCR was performed using an Mx3000P and the 2 × Brilliant III SYBR Green QPCR Master Mix (Agilent Technologies). Each sample was run in triplicate and analysed according to the threshold cycle (Ct) method. For normalization, primer sequences complementary to complementary DNA sequences of *α-tubulin* or Hypoxanthine-guanine-phosphoribosyltransferase (*Hprt*) were used. Primer sequences are summarized Supplementary Table 2.

### Western blot

Pancreatic islets were isolated from control and ILK cKO mice and incubated over night in CMRL medium. On the following day 90 islets were picked in 500 μl CMRL medium and collected by centrifugation at 1,000*g* for 5 min. The islet pellet was lysed in 50 μl ice-cold RIPA buffer supplemented with protease inhibitor (Roche) and phosphatase inhibitor (Roche) and lysis was facilitated using a cell disruptor (Scientific Industries) for 5 min. Finally the lysate was centrifuged at 15,000*g* for 10 min and the supernatant was stored at −80 °C.

Islet lysates were run on Mini-PROTEAN TGX Precast Gels (Bio-Rad Laboratories). Gel electrophoresis was performed with the Mini-PROTEAN Tetra Cell system in combination with the Power Pac Basic power supply (Bio-Rad Laboratories). The proteins were blotted with Trans-Blot Turbo Transfer Packs using the Trans-Blot Turbo Transfer System (Bio-Rad Laboratories). The membranes were cut according to the molecular weight of the examined proteins and pieces individually blocked in a blocking solution with 0.5% Tween 20/PBS and 5% BSA for 1 h. Membranes were incubated over night in blocking buffer with primary antibodies rabbit anti-ILK (1:500, 3862, s, Cell Signaling Technologies) or rabbit anti-GAPDH (1:1,000, ab9485, Abcam) at 4 °C. Secondary antibodies (donkey anti-rabbit conjugated with horseradish peroxidase) were applied for 1 h at room temperature. Membranes were developed with the Clarity Western ECL substrate (Bio-Rad Laboratories) and imaged with the ChemiDoc XRS Imaging System (Bio-Rad Laboratories).

### Pimonidazole injection

To monitor hypoxia in the islets, mice were injected with 60 mg kg^−1^ Pimonidazole (Hypoxyprobe Green Kit, Hypoxyprobe, Inc., Burlingston). Pancreas and liver were subsequently embedded ion OTC and shock frozen. Staining was performed on 12 μm sections that were fixed in aceton at room temperature for 1 min, subsequently washed in PBS 0.2% Tween and blocked in 2% BSA, 5% donkey serum in 0.2% Tween in PBS for 1 h. Sections were stained with HP-FITC-MAb (detects pimonidazole adducts) over night at 4 °C followed by DAPI nuclear counterstaining. For quantification, islets were imaged using an LSM 710 (Zeiss) and images captured with equal exposure times. Intensities of green fluorescence were quantified using Fiji.

### VEGF-A secretion assay

Pancreatic islets were isolated from control and ILK cKO mice and incubated overnight in CMRL medium. On the following day 20 islets were transferred into a single well of a 24- well plate and incubated in 800 μl CMRL medium. After 24 h 500 μl medium were collected and centrifuged at 2,000 r.p.m. for 10 min. One-hundred microlitres supernatant (50 μl duplicates) were used to measure secreted VEGF with the mouse VEGF Quantikine ELISA Kit following the manufacturer's instructions (MMV00, R&D Systems). Subsequently, the islets were lysed in RIPA buffer (50 mM Tris-HCl (pH 7.4), 150 mM NaCl, 1 mM EDTA, 1 mM Na_3_VO_4_, 1 mM NaF, 0.25% Na-deoxycholate, 1% IGEPAL) to measure total protein concentrations using a BCA assay. Secreted VEGF was normalized to total protein levels and presented as a percentage of VEGF secreted from control islets.

### Injection of fluorescent tomato lectin

FITC-conjugated lectin from *Lycopersicon esculentum* (tomato lectin) (Sigma) was intravenously (i.v.) injected into the tail vein of a control and an ILK cKO mouse. Five minutes after injection, mice were killed and whole pancreata were isolated and processed for immunostaining as described above.

### Islet transplantation into the anterior eye chamber

Transplantation of isolated islets into the anterior eye chamber of NOD-Scid immunodeficient mice followed by *in vivo* imaging was performed as previously published^68^,^69^. Briefly, 20–30 handpicked overnight-cultured islets were transplanted into the anterior chamber of the anesthetized 7- to 13-week-old male NOD.CB17-Prkdc scid/J recipient mice using a custom made beveled glass cannula. Longitudinal *in vivo* imaging of transplanted islets was performed at indicated post-transplant days using an upright laser-scanning microscope (LSM780 NLO; Zeiss, Germany). The volume of transplanted islets was assessed by detection of 633 nm laser backscatter. Vessels were visualized by injecting 0.25 mg 500-kDa fluorescein-labelled dextran (Life Technologies) in 100 μl PBS into the tail vein. Fluorescein-labelled dextran was excited by two-photon laser at 910 nm and detected at 493 to 612 nm. Total islet volume and vessel volume were calculated using surface rendering (Imaris 7.6, Bitplane AG, Switzerland).

### Statistical analyses

Throughout the paper, quantifications are shown as means±standard deviations (s.d.). Statistical significance was determined as indicated in the respective figure legends and differences were considered significant with a *P* value<0.05. Normality was visually evaluated (dot plot and Q-Q plots) in addition to the Shapiro–Wilk-test using R and Graphpad Prism. A two-sided, unpaired Student's *t*-test correcting for unequal variances was used for comparison between two groups. For comparisons of more than two groups, one-way ANOVA or two-way ANOVAs were performed. In addition, Dunnett´s or Tukey's multiple comparison test were performed using Graphpad Prism (GraphPad Software). To validate the parametric ANOVA analysis in Fig. 5, a two-way ANOVA with interaction using a quantile regression model for the median (SAS, PROC QUANTREG) and additionally using a robust regression model following the idea of Huber's M-estimation (SAS, PROC ROBUSTREG) was used. In all cases, statistical significance were considered with *P* value<0.05. Most sample sizes were chosen based on data shown in previous publications.

### Electron microscopy

Pancreata were fixed for 2 h at room temperature by immersion in 2.5% glutaraldehyde in 0.19 M sodium cacodylate buffer at pH 7.4, postfixed in 1% reduced osmium tetroxide in aqua bidest for 60 min, and subsequently stained with 2% uranyl acetate in maleate buffer, pH 4.7. The specimens were dehydrated in graded ethanols and embedded in epoxy resin^70^. Ultrathin sections were picked up onto Formvarcarbon-coated grids, stained with lead citrate, and viewed in a transmission electron microscope (TEM 910; Zeiss Elektronenmikroskopie, Oberkochen, Germany).

### Data availability

All relevant data are included in the manuscript or its supplementary information and available from the authors upon request.

## Additional information

**How to cite this article:** Kragl, M. *et al*. The biomechanical properties of an epithelial tissue determine the location of its vasculature. *Nat. Commun.* **7,** 13560 doi: 10.1038/ncomms13560 (2016).

**Publisher's note:** Springer Nature remains neutral with regard to jurisdictional claims in published maps and institutional affiliations.

## Supplementary Material

Supplementary Information Supplementary Figures 1-12 and Supplementary Tables 1-2.

Supplementary Movie 1 Three-dimensional view of the vascular network in a freshly isolated control islet. The islet was stained for insulin (red) and CD31 (green). Z-stack confocal images were taken and projected using the Brightest Point method. Scale bar: 50 μM.

Supplementary Movie 2 Three-dimensional view of the vascular network in a freshly isolated ILK KO islet. The islet was stained for insulin (red) and CD31 (green). Z-stack confocal images were taken and projected using the Brightest Point method. Scale bar: 50 μM.

Supplementary Movie 3 Three-dimensional view of the actin cytoskeleton in a control islet 7 days after plating on fibronectin. The islet was stained for F-actin using a FITC-coupled phalloidin (green). Z-stack confocal images were taken and projected using the Brightest Point method. Scale bar: 50 μM.

Supplementary Movie 4 Three-dimensional view of the actin cytoskeleton in an ILK KO islet 7 days after plating on fibronectin. The islet was stained for F-actin using a FITC-coupled phalloidin (green). Z-stack confocal images were taken and projected using the Brightest Point method. Scale bar: 50 μM.
