# Supplementary material for: The biomechanical properties of an epithelial tissue determine the location of its vasculature
Source: Nat Commun. 2016 Dec 20;7:13560. doi: 10.1038/ncomms13560 (PMC5187430; doi:10.1038/ncomms13560)
Supplement: Supplementary Information — Supplementary Figures 1-12 and Supplementary Tables 1-2. [file ncomms13560-s1.pdf]

Supplementary Information

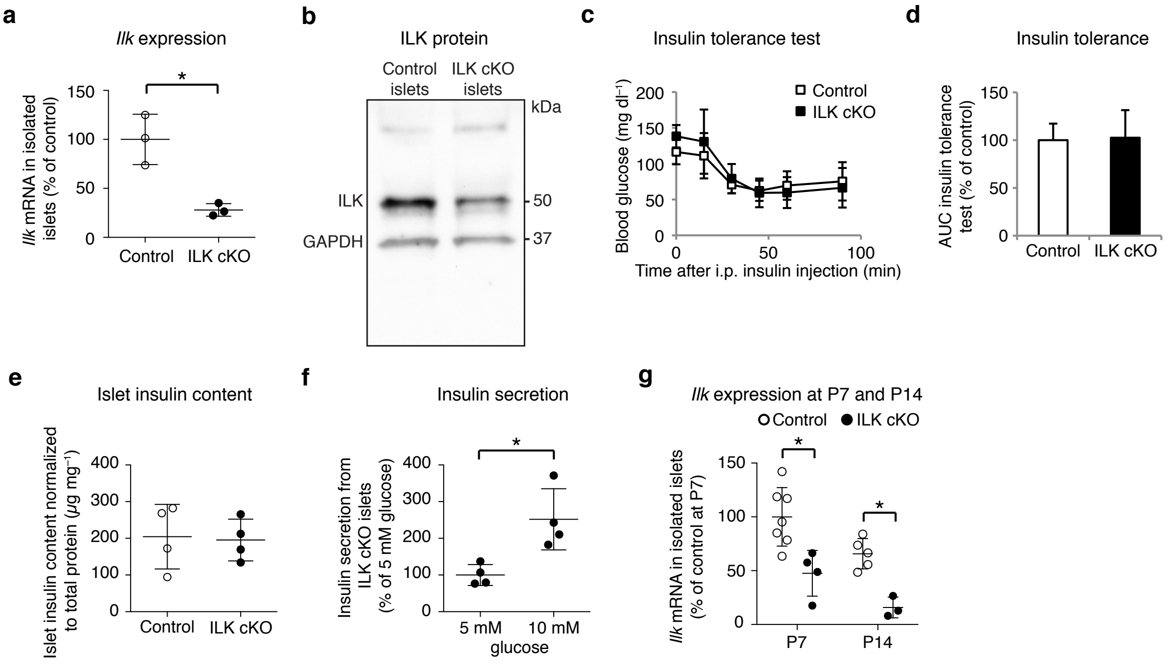

Supplementary Fig. 1: Knockout efficiency and insulin tolerance of ILK cKO mice.
(**a**) Quantification of relative *Ilk* mRNA expression in control versus ILK cKO islets. N = 3 animals per experimental group. (**b**) Western blot of ILK and GAPDH from control and ILK cKO islets. Around 90 islets from two mice were combined to gain lysates for western blot. (**c**) Blood glucose concentrations during an insulin tolerance test in male mice (12 weeks). Insulin (0.75 IU kg^{-1} body weight) was injected at 0 minutes. N = 7-8 mice per experimental group. (**d**) Area under curve of data shown in (c). (**e**) Insulin content from isolated control and ILK cKO islets. N = 4 islet batches per experimental condition (genotype). (**f**) Insulin secretion from isolated ILK cKO islets at 5 and 10 mM glucose. N = 4 islet batches per experimental condition (glucose concentration) each. (**g**) Quantification of relative *Ilk* mRNA expression in control versus ILK cKO islets of 7 (P7) and 14 (P14) day old mice. N = 3-7 animals per experimental group. Data are shown as mean values ± SD, *p < 0.05 in an unpaired, two-sided Student's *t*-test with (c) Holm-Bonferroni correction.

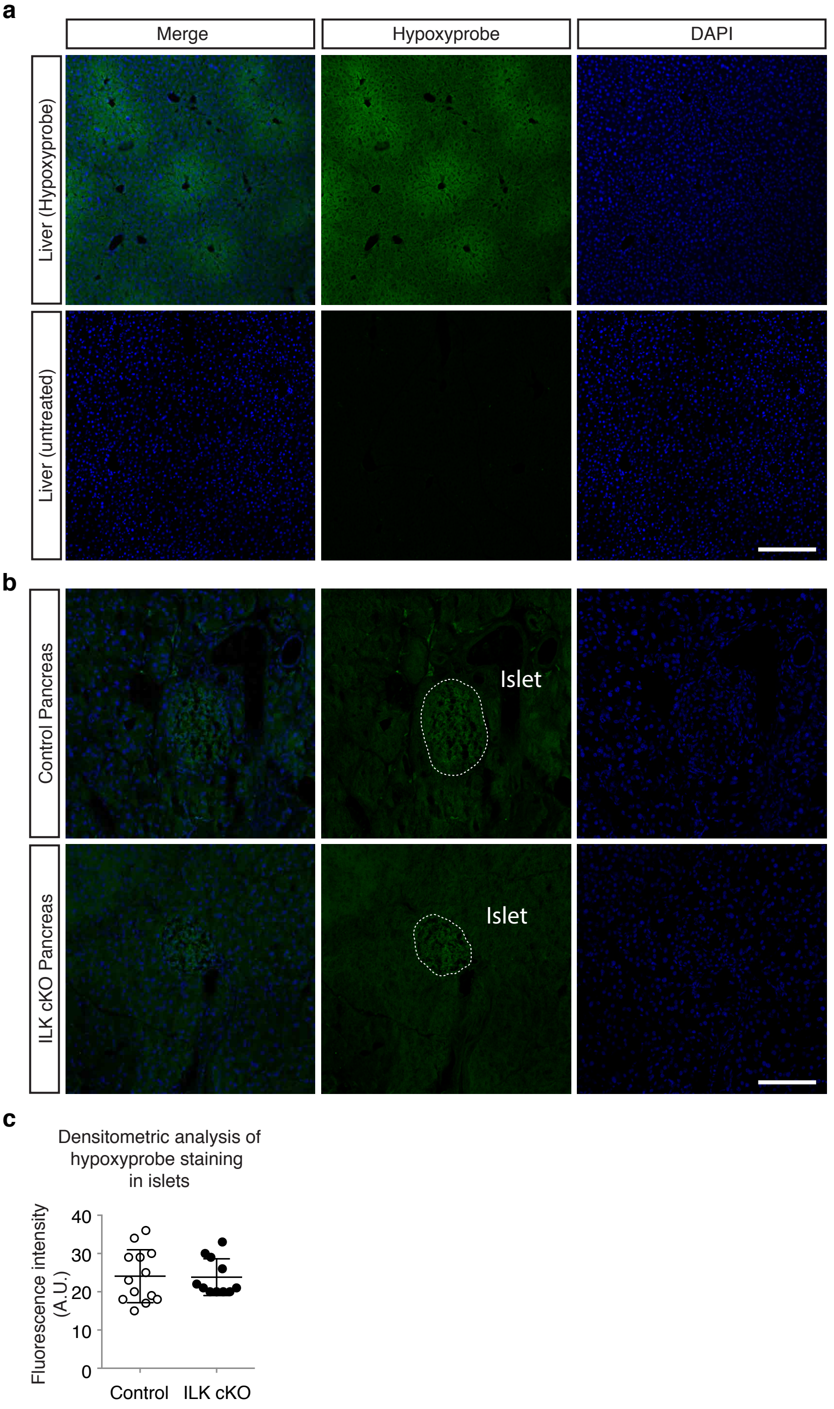

Supplementary Fig. 2: Analysis of hypoxia in islets using pimonidazole (Hypoxyprobe). LSM images of Hypoxyprobe (green) and DAPI nuclear staining (blue) in the (**a**) liver of pimonidazole injected (upper panels) and untreated animals (lower panels) and in the (**b**) pancreas of pimonidazole injected control (upper panels) and ILK cKO mice (lower panels). (**c**) Densitometric quantification of fluorescent intensity in sections of N = 13 control and 11 ILK cKO islets combined from two mice per genotype. Data are shown as mean values ± SD, *p < 0.05 in an unpaired, two-sided Student's *t*-test. Scale bars: (a) 100 µm, (b) 200 µm.

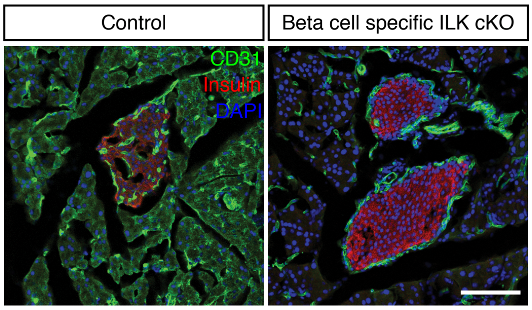

Supplementary Fig. 3: Vascular phenotype in a beta cell specific knockout of *Ilk*.
LSM images of immunofluorescence staining for cell nuclei (DAPI, blue), insulin (red) and CD31 (green) in pancreatic sections from insulin 1 *(Ins1)-Cre; Ilk*$^{loxP/loxP}$ mice and heterozygous control mice. Scale bar: 100 µm.

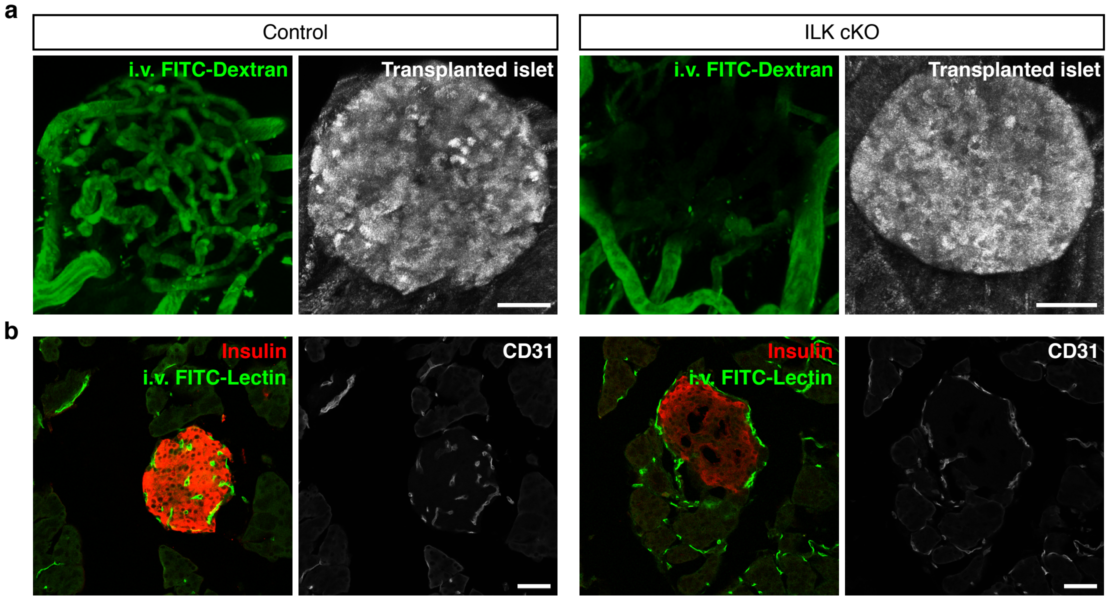

Supplementary Fig. 4: Perfusion of ILK cKO islets after transplantation or *in situ*.
(**a**) *In vivo* images of a control islet and an ILK cKO islet 28 days post transplantation into the anterior eye chamber. The islets are visualized by backscatter and an i.v. injected FITC-conjugated dextran (green) that highlights the perfused blood vessels. (**b**) LSM images showing immunofluorescence staining for insulin (red) of pancreatic sections from a control and an ILK cKO mouse that have been i.v. injected with a FITC-conjugated tomato lectin (green), indicating perfused blood vessels. Scale bars: (a, b) 50 µm.

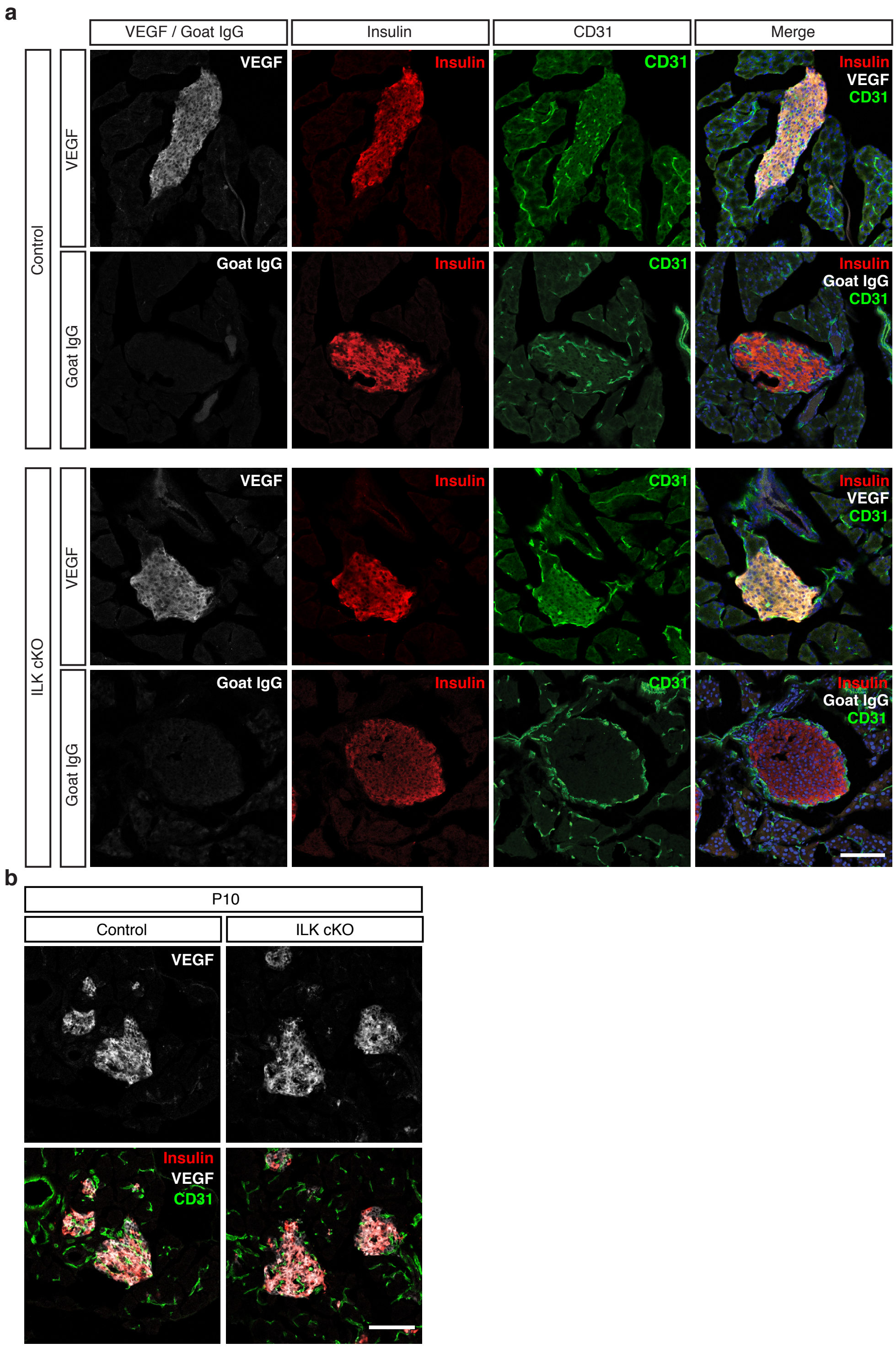

Supplementary Fig. 5: Localization of VEGF-A in ILK cKO pancreas.
(**a**) LSM images of immunofluorescence staining for insulin (red), CD31 (green) and VEGF (white) or Goat IgG (white) in pancreatic sections from adult control and ILK cKO islets. (**b**) LSM images of immunofluorescence staining for insulin (red), CD31 (green) and VEGF (white) in pancreatic sections from 10 days old (P10) control and ILK cKO mice. Scale bars: (a, b) 100 µm.

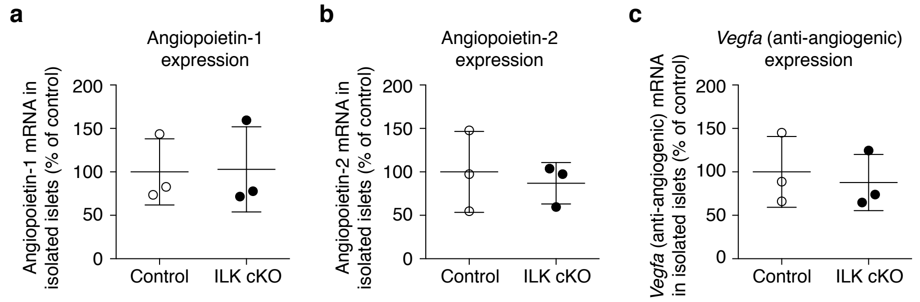

Supplementary Fig. 6: Expression of pro- and anti-angiogenic factors in ILK cKO islets. (**a**) Quantification of relative Angiopoietin-1 mRNA expression, (**b**) relative Angiopoietin-2 mRNA expression, and (**c**) relative anti-angiogenic *Vegf-a* mRNA expression in control versus ILK cKO islets. N = 3 animals per experimental group each**.** Data are shown as mean values ± SD, *p < 0.05 in an unpaired, two-sided Student's *t*-test.

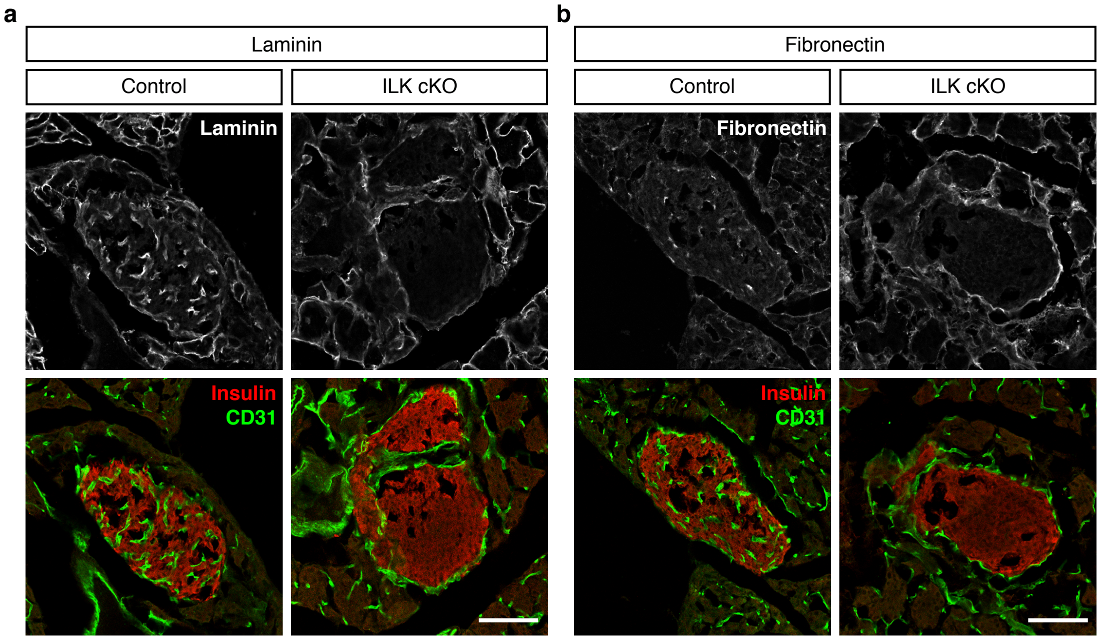

Supplementary Fig. 7: Basement membrane in adult ILK cKO mice.
(**a, b**) LSM images of immunofluorescence staining for laminin (a; white) or fibronectin (b; white), CD31 (green) and insulin (red) in pancreatic sections from adult control and ILK cKO islets. Scale bars: (a, b) 100 µm.

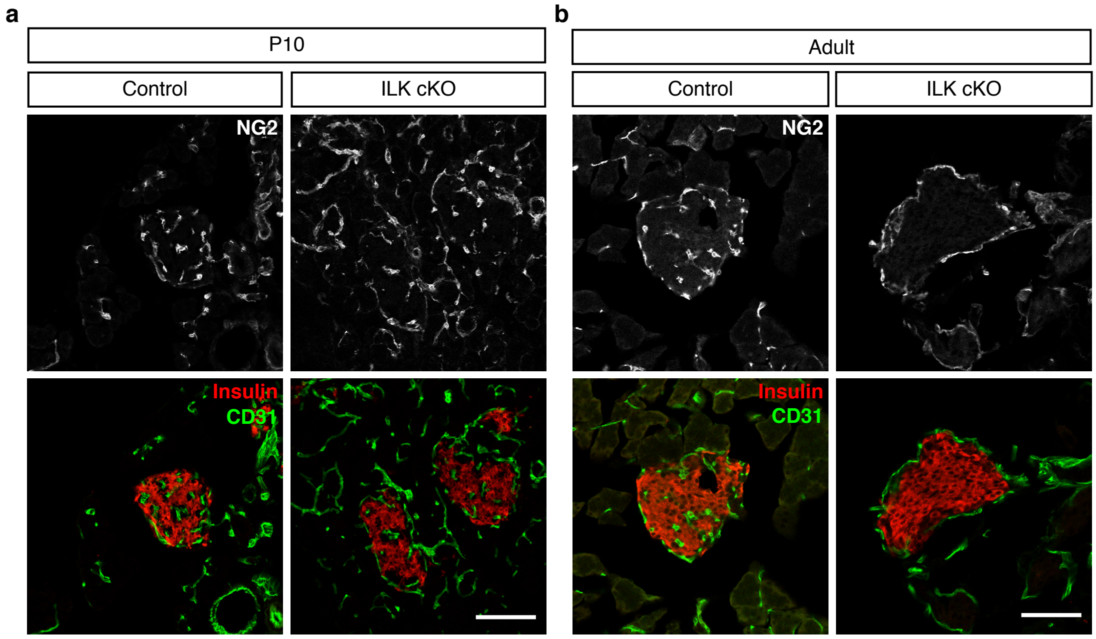

Supplementary Fig. 8: Staining of pericytes in P10 and adult ILK cKO mice.
(**a, b**) LSM images of immunofluorescence staining for pericyte marker NG2 (white), CD31 (green), and insulin (red) in pancreatic sections from control and ILK cKO islets of 10 days (P10) (a) or three week (adult) (b) old mice. Scale bars: (a, b) 100 µm.

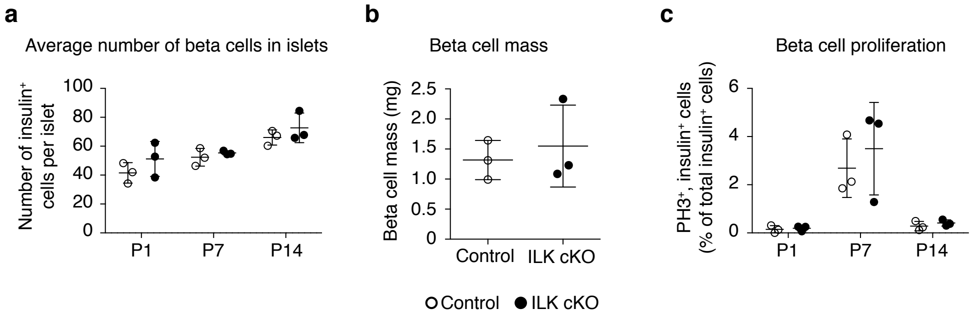

Supplementary Fig. 9: Quantification of beta cell numbers, mass and proliferation. (**a**) Quantification of the average number of beta cells in islets at P1, P7 and P14. N = 3 animals per experimental group. Clusters of ≥ 10 insulin$^+$ beta cells were considered as islets for the quantification. 28-39 (P1), 53-114 (P7) and 47-77 (P14) islets were analyzed per animal. (**b**) Beta cell mass per mouse in control and ILK cKO mice. N = 3 mice per genotype. (**c**) Quantification of proliferating pancreatic beta cells. Insulin$^+$, PH3$^+$ cells are shown as percentages of the total number of insulin$^+$ cells in islets of pancreatic sections from mice at the age of 1, 7 and 14 days. N = 3 animals per experimental group. A total number of 933-2,319 (P1), 2,646-4,852 (P7) and 2,976-5,183 (P14) insulin$^+$ beta cells were analyzed per animal. Data are shown as mean values ± SD, *p < 0.05 in an unpaired, two-sided Student's *t*-test.

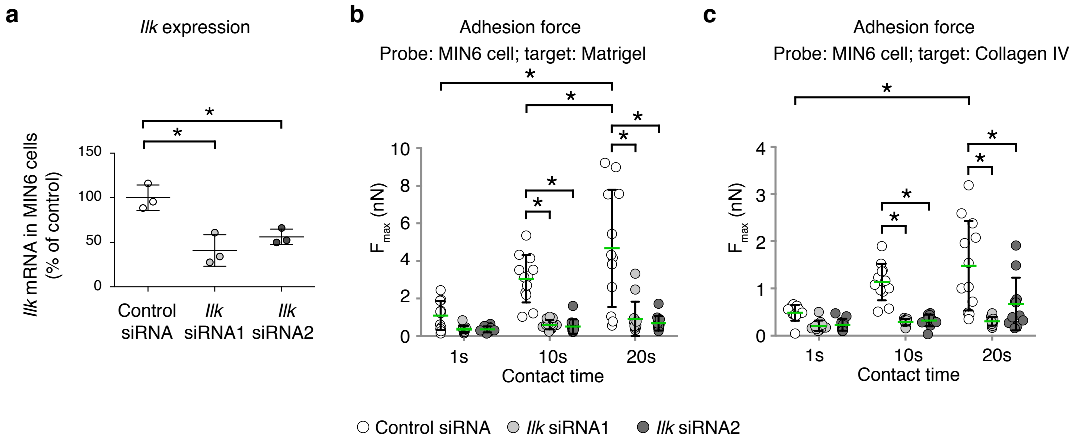

Supplementary Fig. 10: Requirement of ILK for adhesion to basement membrane. (**a**) Quantification of relative *Ilk* mRNA expression in MIN6 cells, a mouse insulinoma cell line treated with either a control siRNA or two siRNAs against *Ilk*. (**b,c**) SCFS measurement of the adhesion force between MIN6 cells (probe), treated with either control or two *Ilk* siRNAs, and matrigel (b) or collagen IV (c) as targets. N = 10-13 MIN6 cells versus matrices were tested per experimental condition (contact time and siRNA treatment). The green lines indicate average values. Data are shown as mean values ± SD, *$p < 0.05$ in a one-way ANOVA followed by Dunnett's multiple comparisons test (a) or a two-way ANOVA followed by Tukey's multiple comparisons test (b, c).

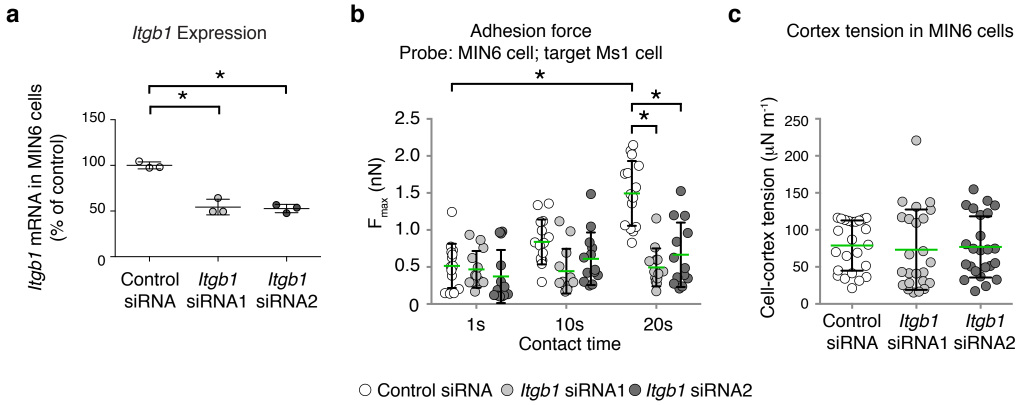

Supplementary Fig. 11: Adhesion to Ms1 endothelial cells requires β1 integrin.
(**a**) Quantification of relative β1 integrin (*Itgb1)* mRNA expression in MIN6 cells treated with either a control siRNA or two siRNAs against *Itgb1*. (**b**) SCFS measurements of adhesion force between MIN6 cells (probe), treated with either control or two *Itgb1* siRNAs, and Ms1 endothelial cells (target). N = 13-15 MIN6 cells versus 13-15 different Ms1 cells were tested per experimental condition (contact time and siRNA treatment). The green lines indicate average values. (**c**) Colloidal force microscopy measurements of cortex tension in MIN6 cells treated with either a control or two different *Itgb1* siRNAs. N = 24 cells were tested per experimental condition (siRNA). Data are shown as mean values ± SD, *$p < 0.05$ in a one-way ANOVA followed by Dunnett's multiple comparisons test (a), a two-way ANOVA followed by Tukey's multiple comparisons test (b) or a one-way ANOVA followed by Tukey's multiple comparisons test (c).

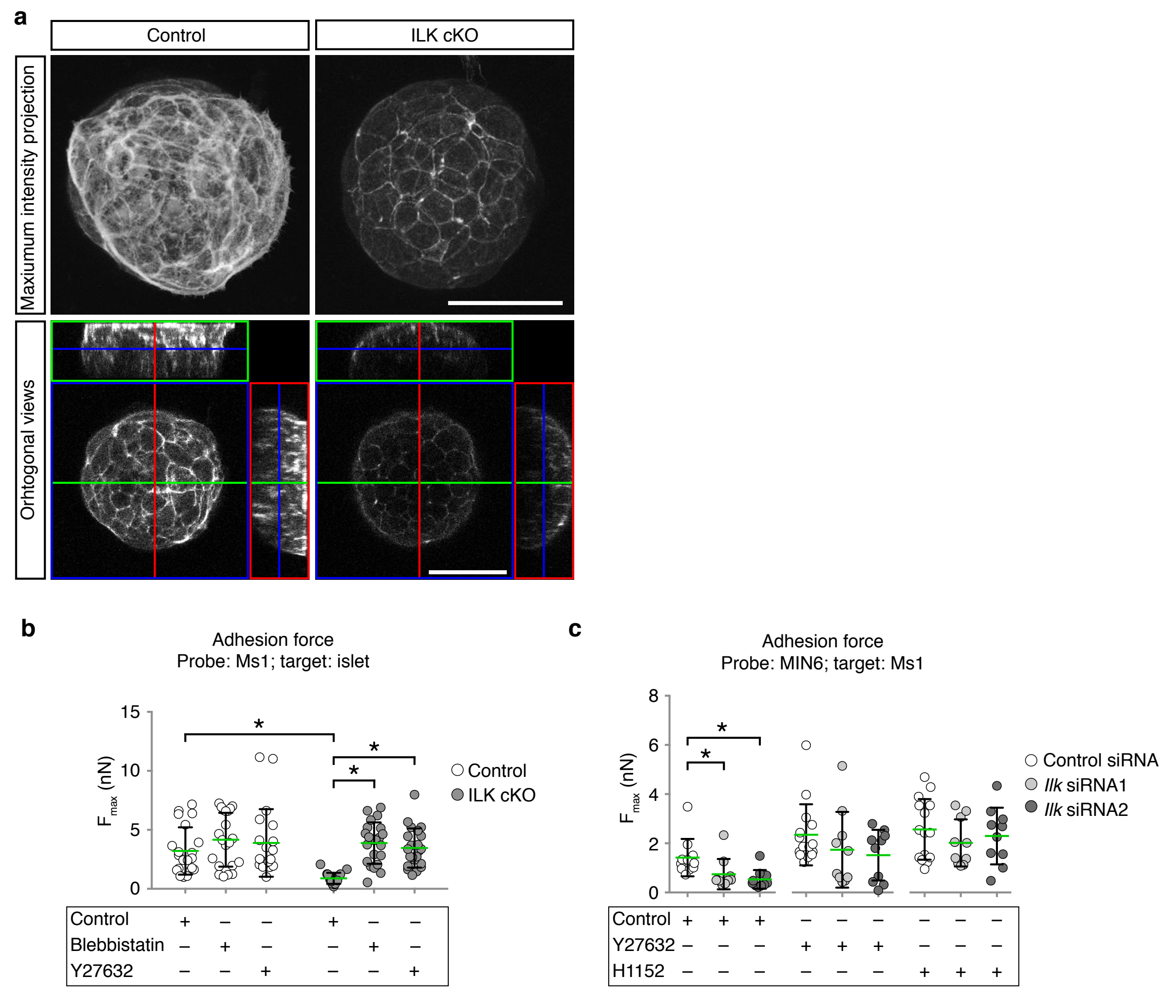

Supplementary Fig. 12: ILK dependent adhesion to fibronectin and Ms1 endothelial cells via cortical actomyosin.

(a) Maximum intensity projection images of LSM z-stacks from F-actin staining of representative control and ILK cKO islets 7 days after plating on fibronectin. Orthogonal views of LSM z-stacks of F-actin-stained control and ILK cKO islets (blue box). The view in XY plane is presented in the green box and XZ plane the red box. (**b**) SCFS measurements of adhesion forces between Ms1 endothelial cells (probe) and ILK cKO versus control islets (targets) after a contact time of 5 minutes, with or without blebbistatin or Y27632 treatment prior to the experiment. N = 20-22 islets were tested for each condition (genotype ± blebbistatin ± Y27632). The green lines indicate average values. (**c**) Adhesion forces between MIN6 cells (probe) and Ms1 cells (target) after a contact time of 20 seconds, with or without treatment with Y27632 or H1152 (both inhibitors of ROCK) prior to the experiment. N = 10-15 MIN6 cells versus 10-15 different Ms1 cells were tested per experimental condition (siRNA, ± Y27632, ± H1151). Data are shown as mean values ± SD, *$p < 0.05$ in a two-way ANOVA followed by Tukey's multiple comparisons (b) or a one-way ANOVA followed by Tukey's multiple comparisons (c). Scale bar: (a) 50 µm.

Supplementary Tables

Table 1. Islet endothelial cell apoptosis

		Total ECs	Apoptotic ECs
P7	Control 1	75	1
	Control 2	309	0
	Control 3	269	0
	ILK cKO 1	132	0
	ILK cKO 2	334	0
	ILK cKO 3	193	0
P10	Control 1	300	0
	Control 2	161	0
	Control 3	239	0
	ILK cKO 1	95	0
	ILK cKO 2	104	1
	ILK cKO 3	346	0

Quantification of cleaved caspase-3^{+} vascular endothelial cells in control and ILK cKO islets from mice at the age of 7 days (P7) and 10 days (P10). N = 3 animals per experimental group.

Table 2. Primer sequences for real-time RT-PCR

Gene	Sequence
mouse *Ilk* forward	5'-GTGGCTGGACAACACAGAGA-3'
mouse *Ilk* reverse	5'-ATCCCCACGATTCATCACAT-3'
mouse α-tubulin forward	5'-AGAGTCGCGCTGTAAGAAGC-3'
mouse α-tubulin reverse	5'-TCTTCTCACTTGGCATCTGG-3'
mouse *Hprt* forward	5'-CACAGGACTAGAACACCTGC-3'
mouse *Hprt* reverse	5'-GCTGGTGAAAAGGACCTCT-3'
mouse *Vegfa* forward	5'-CAAGATCCGCAGACGTGTAA-3'
mouse *Vegfa* reverse	5'-TTAATCGGTCTTTCCGGTGA-3'
mouse "antiangiogenic" *Vegfa* forward	5'-GGACACATTAGGGGGTGTTG-3'
mouse "antiangiogenic" *Vegfa* reverse	5'-GGTGAGAGGTCTGCAAGTACGTT-3'
mouse Angiopoietin 1 forward	5'-CAGCACGAAGGATGCTGATA-3'
mouse Angiopoietin 1 reverse	5'-ATGGTGGTGGAACGTAAGGA-3'
mouse Angiopoietin 2 forward	5'-GCACAAAGGATTCGGACAAT-3'
mouse Angiopoietin 2 reverse	5'-ATCATGGTTGTGGCCTTGAG-3'
mouse *Itgb1* forward	5'-AATGCCAAGTGGGACACGGG -3'
mouse *Itgb1* reverse	5'-TGACTAAGATGCTGCTGCTGTGAGC-3'
